# Multiplexed polypeptide-based hybrid bacterial clusters by tailoring the conjugation for synergistic treatment of infected wounds

**DOI:** 10.1016/j.mtbio.2025.102040

**Published:** 2025-07-01

**Authors:** Jiang Xiao, Zhongquan Song, Xiangdong Lai, Xiangyang Zhang, Xiaohui Liu, Hui Jiang, Minjie Li, Xuemei Wang

**Affiliations:** a State Key Laboratory of Digital Medical Engineering, School of Biological Science and Medical Engineering, Southeast University, Nanjing, 210096, China; b Key Laboratory for Quality Evaluation of Bulk Herbs of Hunan Province, School of Pharmacy, Hunan University of Chinese Medicine, Changsha, 410208, Hunan, China; c Zhongda Hospital, Medical School, Southeast University, Nanjing, 210009, China; d Furong Laboratory, Changsha, 410208, Hunan, China

**Keywords:** Bacterial surface modification, Polypeptide, Bionic assembly, Promote wound healing, Antibacterial

## Abstract

Drug-resistant bacterial infections and excessive inflammation pose serious challenges to wound healing. Currently, biomaterial-assisted antibacterial therapies demonstrate excellent therapeutic potential by enriching antibacterial agents at the site of infection and combining the advantages of multiple therapeutic agents. Here, inspired by bacterial aggregation behaviors in living organisms that regulate host physiological activities, a biomimetic polypeptide-based hybrid bacterial cluster is reported for treatment of infected wounds (PTS-UGT). Specifically, hybrid bacteria (UGT) were first constructed by a method of stepwise biological self-assembly with Staphylococcus epidermidis (SE) bacterial surface growth of gold and silver particles (UG) and polyphenolization of gold and silver particles. Subsequently, under the inducement of borate-diphenol coordination interactions and multiple hydrogen bonding, UGT was assembled with the side-chain boronic-acid-modified antibacterial polypeptide tobramycin (PTS) to form biomimetic hybrid bacterial clusters (PTS-UGT) by tailoring the conjugation methods. These clusters integrate the multiplexing and aggregation enhancement functions of bio-self-assembled gold, silver, polyphenol particles, bacterial immunomodulatory components and antibacterial polypeptides, which can effectively regulate the polarization of macrophages to M2 type and enhance the local effective drug concentration for multifunctional bacterial killing, thereby showing excellent antibacterial, anti-inflammatory and healing effects on infected wounds in mice, providing new ideas for the development of biomaterial-assisted antibacterial therapy.

## Highlights

• Drawing inspiration from the close influence of bacterial spatial aggregation on host-microbe interactions, the polypeptide-based hybrid bacterial cluster (PTS-UGT) was designed for synergistic treatment of infected wounds.
• The bacteria cluster (PTS-UGT) was constructed through a straightforward method of stepwise biological self-assembly of Staphylococcus epidermidis and tailoring the conjugation based on boric acid-polyphenol-metal ion interaction.
• This study proposed a strategy of engineering hybrid commensal bacteria with multiplexing and aggregation-enhancing functions for antibacterial and anti-inflammatory.

## Graphical abstract

Inspired by the close influence of bacterial spatial aggregation on host-microbe interactions, the multiplexed polypeptide-based hybrid bacterial cluster (PTS-UGT) with antibacterial and anti-inflammatory efficacy was constructed through a straightforward method of stepwise biological self-assembly and tailoring the conjugation based on boric acid-polyphenol-metal ion interaction for synergistic treatment of infected wounds.

**Figure:**
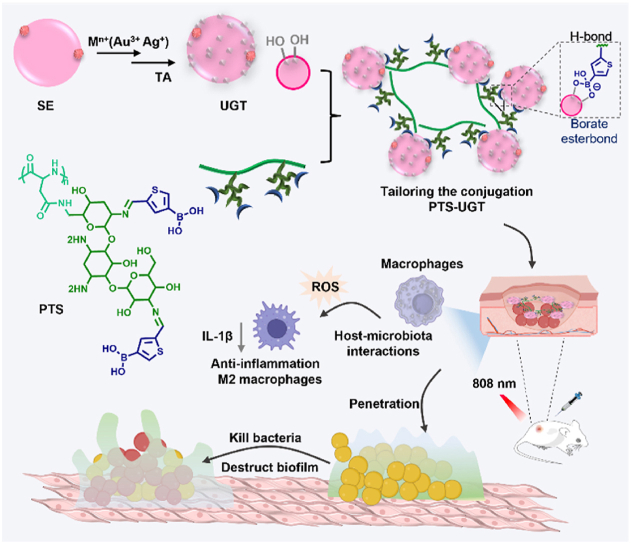

## 1 Introduction

Microbial Drug-resistant bacterial infections and excessive inflammation are major factors that delay wound healing and will even lead to severe chronic skin wounds, as well as serious complications that are life-threatening [1]. Currently, antibiotic therapy is the mainstay of clinical antibiotic therapy. However, the widespread use of antibiotics has led to bacterial resistance, prompting a large number of novel non-antibiotic agents for alternative therapies, such as metal nanomaterials, antibacterial peptides, biologic therapies, and photothermal therapies [[2], [3], [4], [5], [6]].

Biomaterial-assisted antibacterial therapies show good clinical therapeutic promise, help to enrich antibacterial agents at the site of infection, and can be combined with multiple therapeutic modalities, which in turn enhances efficacy [[7], [8], [9], [10], [11], [12], [13], [14]]. Among them, functionalized hybrid bacterial systems using chemical surface modification, which combine excellent properties such as bioactivity modulation ability, optical and electrochemical properties of inorganic nanomaterials and physicochemical properties of drugs, are being explored for the treatment of cancer, intestinal and lung inflammation, as well as bacterial infections diseases [[15], [16], [17]]. The microbial-assisted synthesis of metal nanoparticles is simpler and safer. After metal ion species selection and synthesis optimization, it can have excellent host-microbiome interactions and achieve two-component metal synergistic effects. It is one of the ideal strategies for constructing hybrid bacteria with antibacterial and anti-inflammatory effects [[18], [19], [20], [21]]. However, the efficacy of hybrid bacterial drug systems based on bacterial engineering still needs to be improved due to environmental susceptibility and limited accumulation at the site of disease [22].

Drawing inspiration from clustering behavior in nature, researchers precisely arrange nanohybrid materials and micro-nano robots in order to improve the physical and chemical properties of the materials [23]. For example, artificial biofilms prepared by co-assembly of graphene oxide and bacteria can greatly enhance the generation of electric current [24]. Recent studies have shown that the spatial organization and aggregation degree of gut microbiota, such as bacterial clusters (or colonies), varies in morphology and spatiotemporal aggregation, which is closely related to host-microbe interactions [[25], [26], [27], [28], [29]]. Therefore, drawing on these inspirations, we envisioned constructing bacterial aggregates (i.e., hybrid bacterial clusters) with multiplexing and assembly synergy to explore their aggregation-potentiating effects in resisting bacterial infection and regulating host life functions [30,31], especially their therapeutic potential in targeting infected wound healing [32,33].

To meet this vision, a skin commensal, Staphylococcus epidermidis (SE), which has the ability to immunomodulate and inhibit infections, was used to construct bionic hybrid bacterial clusters [[34], [35], [36]]. Polypeptides have both the biomimetic regulatory functions of biomimetic peptides and the potential advantages of large-scale synthesis. For example, side-chain phosphorylated and borylated polypeptides can induce biomineralization and aggregation tendency on the cell membrane surface, respectively. Therefore, this study applied them to the bridging design of hybrid bacterial clusters [33,37]. Meanwhile, for the construction of hybrid bacterial clusters with aggregation potentiation, the use of a drug-specific coupling approach should be a feasible way of potentiation. Briefly speaking, the drug coupling rate is controlled for surface-modified bacteria, and the effective components on the bacterial surface are appropriately exposed to avoid the formation of drug all-encapsulation. By this strategy, the multiplexing of each component of the hybrid bacteria clusters is achieved, and the multifunctional synergistic therapeutic effect of multicomponent hybrid bacterial clusters containing antibacterial drugs, nanoparticles, and bioactive components of symbiotic bacteria can be realized [24,[38], [39], [40]].

**Scheme 1:**
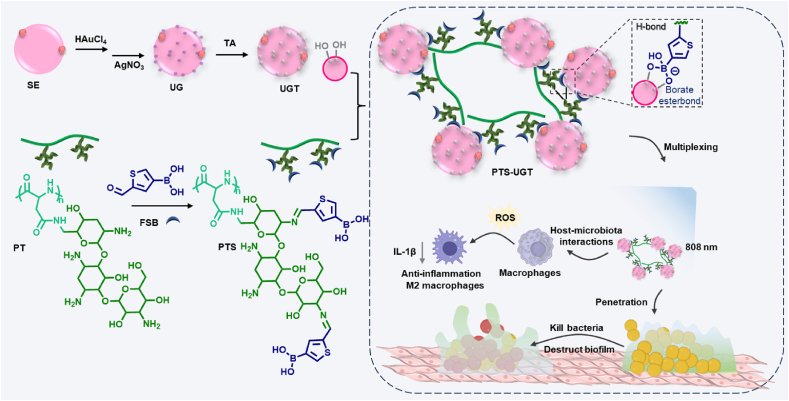
Schematic illustration of constructing biomimetic polypeptide hybrid bacterial clusters to antibacterial and reduce inflammation for synergistic treatment of infected wounds.

In this paper (Scheme 1), we first used the biological self-assembly method to prepare surface gold and silver hybrid bacteria (UG) with potential bimetallic synergistic effects, followed by the addition of anti-inflammatory drug tannic acid to further reduce bacterial surface gold and silver to allow the anchorage of polyphenol sites (UGT) at the bacterial gold and silver surface [41]. Next, the UGT were co-assembled with tobramycin (TOB) functionalized polypeptide (PT) containing boric acid moieties in the side chain (PTS) by the boric acid-polyphenol coordination and multiple hydrogen bonding [25], to construct the antibacterial bionic hybrid bacterial clusters (PTS-UGT). As a proof-of-concept, the polypeptide-based gold and silver hybrid bacterial clusters can mimic the microbial aggregation effect with enhanced photothermal and electrochemical activity, inhibition of excessive inflammatory hyperplasia, and the ability to efficiently remove bacteria and their biofilms, which in turn exhibited excellent pro-healing synergistic treatment effects on bacterial infected wounds in mice.

## 2 Experimental

### 2.1 Synthesis of PT and Cy5-Labeled PT (PT-Cy5)

PBLA (0.5 g, 4.25 mmol) or PBLA-Cy5 was dissolved in 25 mL of DMSO, followed by the addition of 3 equivalents of Tobramycin.The reaction mixture was allowed to react for 36 h at room temperature for the amine ester exchange reaction. The reaction solution was then dialyzed in deionized water for 3 days (water changed three times daily). The resulting white powder product was obtained after freeze-drying (yield: 42 %).

### 2.2 Synthesis of UGT

The freshly cultured Staphylococcus epidermidis were resuspended in PBS, added with HAuCl_4_ solution at a final concentration of 150 μM and shaken for 4 h at 37 °C. After centrifugation and washing with PBS twice, the sample was added with AgNO_3_ solution at a final concentration of 150 μM and continuously shaken for 4 h to obtain UG. To prepare UGT, the UG was added with TA solution at a final concentration of 150 μM and shaken for 4 h, then centrifuged and washed twice, and resuspended in PBS for storage.

### 2.3 Synthesis of PTS-UGT and Cy5-Labeled PTS-UGT (PT-Cy5-UGT)

A sufficient amount of PT or PT-Cy5 (final concentration = 1 mg/mL) and 5-formyl-2-thiophene boric acid (2.2 equivalents of PT or PT-Cy5) were mixed in PBS at room temperature for 2 h. Then UGT (OD_final_ = 0.3) were mixed and shaken at room temperature for a certain time (2 h or 10 h), then centrifuged and washed twice to prepare PTS-UGT, which was resuspended in PBS for storage or dilute to different concentrations for later use (The final concentration is 0.3 OD, which is recorded as 1 equivalent (1 eq)). PTS-UG and Cy5-Labeled PTS-UG (PT-Cy5-UG) was prepared by similar method.

### 2.4 Electrochemical analyses

A three-electrode system was used for CV analysis. According to the literature, 8 μL of each sample solution was dropped on the glassy carbon electrode as a working electrode, and dried at 37 °C. Platinum wire was used as a counter electrode and Ag/AgCl as a reference electrode. The scanning rate was 1 mV/s and was repeated for four cycles in the potential window from - 0.7 V to +0.7 V.

### 2.5 Raman spectroscopic analyses

The sample were mixed with crystal violet at a final concentration of 10^−5^ M and dripped on the substrate. The SERS enhancement effect was detected under the laser excitation of 785 nm.

### 2.6 Phenotypic regulation of macrophages

The macrophage RAW 264.7 cells after 24 h incubation with different samples were washed and fixed, blocked with 5 % BSA for 30 min, incubated with antibodies (anti-CD86-FITC, anti-CD206-PE, and anti-F4/80-APC) for 1 h. The phenotype of macrophage RAW 264.7 cells was analyzed by flow cytometry.

### 2.7 Cell scratch test

The L929 cells were inoculated in a 6-well plate and scratched and washed after the cells covered the bottom of the plate (a gap was made in the center of the well with a 200 μL pipette tip). Then it was replaced by a serum-free DMEM medium containing different sample groups, and observed and photographed under a microscope at different times.

### 2.8 Bacterial killing effect in vitro

Different sample groups were incubated with freshly cultured MRSA at 37 °C for 24 h. Then, the appropriately diluted bacterial suspension of each group (100 μL) was spread on LB agar plates and cultured at 37 °C until mature colonies were formed, and then counted to evaluate the antibacterial properties of the materials.

### 2.9 Penetration and disruption of mature biofilms

Initially, the LB culture containing MRSA was placed in a 6-well plate and incubated at 37 °C to form mature bacterial biofilm. Subsequently, different samples containing Cy5 dye-labeled polypeptide components were applied to each well. After 30 min, the supernatant was slowly aspirated and the biofilm were washed gently and stained with 0.01 % acridine orange for 20 min. Finally, the excess dye was washed off, and the biofilm penetration effect was evaluated by observing and photographing under a CLSM. Furthermore, different samples (Control (PBS), Control + NIR, PTS, PTS-UG, PTS-UGT and PTS-UGT + NIR) were applied to each well, and after overnight treatment, the supernatant was slowly aspirated and washed gently. Finally, the destruction of mature biofilm was observed under a CLSM according to the instructions of the Calcein/PI Live/Dead Viability/Cytotoxicity Assay Kit.

### 2.10 In vivo treatment of bacterial wound infections

First, the mice were anesthetized, and a circular wound with a diameter of approximately 1 cm^2^ was created on the back. Then, MRSA (100 μL, 1 × 108 CFU/mL) was implanted into the wound and allowed to infect for 12 h before conducting the treatment experiment. The mice with infected wounds were divided into six groups, including Control (PBS), Control + NIR, UG, UGT, PTS-UGT, and PTS-UGT + NIR, respectively. After different treatments on day 1, the wound size was periodically photographed, and the mice's weight was recorded up to 10 days. On day 3, wound fluid was collected from the mice for bacterial quantification using a culture plate, and blood samples were collected from the mice to measure the concentrations of TNF-α and IL-1β cytokines. In addition, wound tissues were collected and flow cytometry was used to evaluate the differentiation of macrophages and T cells. On day 10 the mice's wound skin was collected and flow cytometry was used to evaluate the differentiation of macrophages, and stained with hematoxylin-eosin (H&E) and Masson to evaluate the wound healing progress, besides the main organs were collected with H&E staining for the biosafety of PTS-UGT in vivo.

### 2.11 Statistical analysis

All statistical analyses were performed in GraphPad 9.0 (Prism) and Microsoft Excel. All statistical tests were specified in the figure legends. A significant p-value represents a significant difference set at ∗p < 0.05, ∗∗p < 0.01, ∗∗∗p < 0.001, ∗∗∗∗p < 0.0001, n.s means no significance.

## 3 Results and discussion

### 3.1 Construction of biomimetic polypeptide hybrid bacterial clusters

**Fig. 1:**
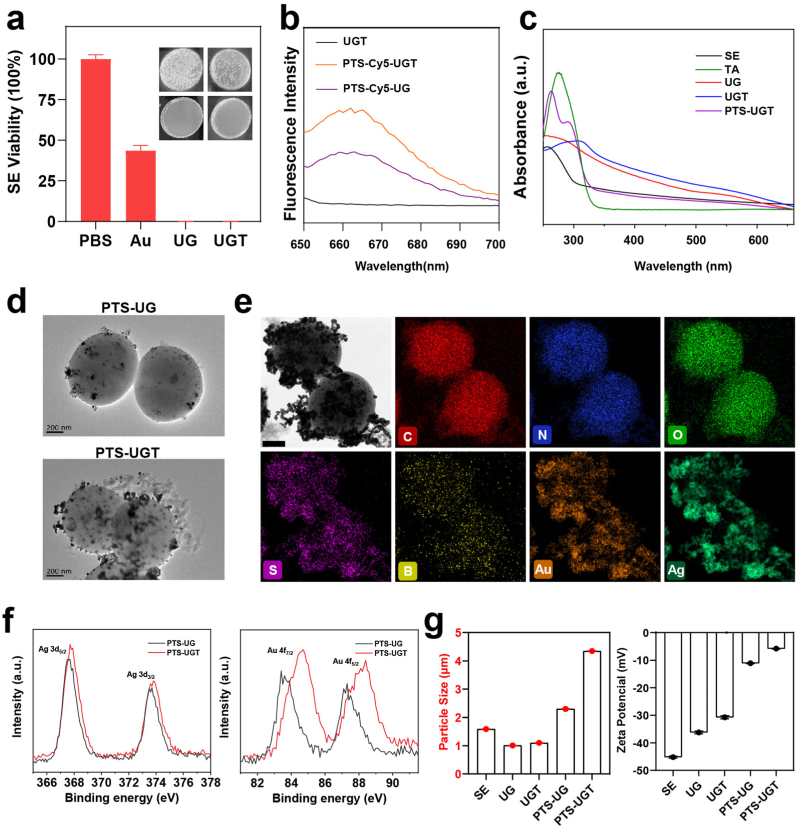
(a) Photographs of plate counts and corresponding statistical histograms after incubation of SE with PBS, Au, UG and UGT, respectively. (b) Fluorescence spectra of UGT, PTS-Cy5-UGT and PTS-Cy5-UG under Cy5 fluorescence excitation wavelength (645 nm). (c) UV–Vis absorption spectra of SE, UG, UGT and PTS-UGT. (d) TEM images of PTS-UG and PTS-UGT. (e) Elemental mapping image of PTS-UGT (Scar bar = 200 nm). (f) XPS of Au and Ag elements in PTS-UG and PTS-UGT. (g) Corresponding particle size distribution and zeta potentials of SE, UG, UGT, PTS-UG and PTS-UGT, respectively.

According to the literature [42], we first prepared hybrid bacteria growing gold and silver on the surface by using the biological self-assembly method. In a representative synthesis method, HAuCl_4_ and AgNO_3_ sequentially in solution were reduced on the surface of Staphylococcus epidermidis (SE), exhibiting a purplish-red colour (UG). Subsequent addition of polyphenolic tannic acid (TA) further reduced the gold and silver components while providing phenolic hydroxyl sites (UGT) in the bacterial surface layer, with the colour of the reaction solution further deepened [43,44]. Meanwhile by plate counting method, it can be seen that compared to the reduced bacterial activity after the addition of chloroauric acid alone, the UG and UGT of the hybrid bacteria after the addition of the multicomponent have completely lost their activity, similar to the inactivated bacterial vaccine (Fig. 1a) [45].

Next, in order to achieve and justify the tailoring the conjugation between hybrid bacteria, We first designed and synthesized side-chain TOB functionalization and terminal fluorescent Cy5-labeled poly-L-aspartic acid derivatives (PT and PT-Cy5) according to the general ring-opening polymerization method for synthesizing polypeptides in the literature [[46], [47], [48]]. TOB was chosen due to the fact that it is an antibacterial drug containing a large number of hydroxyl and amine groups in its molecular structure, and its modification by boronic acid modification can provide more action binding sites for inducing bacterial aggregation (Scheme S1). The molecular structure was verified using ^1^H NMR, while the polypeptide polymerization degree was calculated to be 21, based on the terminal group method such as peak “e” at 1.5–2 ppm (Fig. S1 and S2).

Subsequent side-chain boric acidification of PT was carried out by Schiff base reaction between the aldehyde group and the amino group (Scheme S2). The ^1^H NMR spectrum proved that it was successfully synthesized and has a high graft rate. For example, PT showed that the phenyl peak with chemical shift of 7–8 ppm disappeared, and PTS showed that a new peak with chemical shift of 8–9 ppm appeared (Fig. S3 and S4). The most initial attempts were made to fully introduce 5-formyl-2-thiopheneboronic acid (FSB) into the reaction system, but as a result, after several hours of reaction of the system (e.g.,12 h, PTS(12 h)), probably due to the decrease in the water solubility of the product and the extreme increase in intermolecular hydrogen bonding interactions, a large number of yellow precipitates insoluble in water precipitated, with very low solubility even under acidic conditions and in the presence of trypsin [49,50]. An in situ reaction-induced self-assembly was chosen for the formation of PTS, and subsequent assembly and aggregation to form PTS-UGT, which were induced by the boronic acid-polyphenol-metal ion backbone (Scheme S3). The representative synthesis was specified as follows: firstly, PT was first premixed with FSB (2.5-fold) for 2 h, leading to the formation of acylhydrazone bonds and boronic acid groups in the side chains. Subsequently, UGT was added and co-incubated for 2 h. The aggregation and assembly into PTS-UGT was induced by the borate-phenolic coordination on the surface of the hybrid bacterial and by intermolecular hydrogen bonds.

The fluorescence spectra showed that PTS-Cy5-UGT possessed higher Cy5 fluorescence intensity compared with UGT and PTS-Cy5-UG (Fig. 1b). The UV–Vis spectra showed an absorption peak at around 540 nm for UG, indicating the successful biosynthesis of gold and silver nanoparticles on the surface layer of UG (Table S1), whereas the absorption peak of UGT at around 540 nm showed weaker signals, which predicted that the gold and silver nanoparticles on the surface layer of UGT were further reduced by the polyphenols, with altered sizes and surface properties [51]. In addition, PTS-UGT had new absorption peaks of polypeptides at wavelengths of about 263 nm and 290 nm (Fig. 1c). These results indicated that it provided a large number of phenolic hydroxyl sites on the polyphenol-treated hybrid bacterial surface layer to achieve efficient bioconjugation of polypeptides. In addition, upon Cy5 fluorescence intensity assessment, we chose 100 μg/mL of PT or PTS (much higher than the polypeptide content in PTS-UGT) as one of the control groups for subsequent experiments.

The morphological features during the stepwise preparation of the bionic hybrid bacterial clusters were preliminarily demonstrated by scanning electron microscopy (SEM) (Fig. S5a) and transmission electron microscopy (TEM) (Fig. 1d). TEM image showing small particles of gold and silver nanoparticles by biosynthesis on the surface of SE scaffolds with a size of about 11 nm (Fig. S5b). Compared with PTS-UG, more polypeptide components were coupled at the bacterial surface layer on PTS-UGT, which bridged the hybrid bacteria to form the cluster structure. The element mapping diagram confirmed the existence and overlap of Au, Ag and B in PTS-UGT, and the atomic percentages of Au and Ag are shown in Table S1. It indicates that there is a possible presence of Ag layer on Au seeds, and the bridging of polypeptides on the surface of bacterial clusters by tailoring the conjugation methods (Fig. 1e). The XPS results showed that compared to PTS-UG, the Au4f peaks of PTS-UGT locate at 84.7 and 88.1 eV, with a large shift of 1.0 and 0.9 eV, which indicated that the Au nanoparticles exhibit 0-valence and 1-valence after further reduction by TA (Fig. 1f), and the Ag 3d peaks of PTS-UGT with a shift of 0.1eV exhibit a quinone-based electron acceptor-induced electron-deficient state. The dynamic light scattering (DLS) measurement also showed an increase in the hydrodynamic size of PTS-UGT to ∼4.2 μm, along with a significant change in zeta potential (Fig. 1g and S6). Atomic force microscope (AFM) image analysis shows that the size and surface roughness of PTS-UGT are significantly increased compared with that of SE (Fig. S7 and S8). SDS polyacrylamide gel electrophoresis (SDS-PAGE) results showed that the biomimetic modified PTS-UGT still retained membrane protein bands similar to SE (Fig. S9). The series of characterizations illustrated the successful preparation of biomimetic polypeptide-based hybrid bacterial clusters using tailoring the conjugation and in situ reaction-induced self-assembly approaches.

### 3.2 Optical/electrochemical properties and photothermal effect of PTS-UGT

**Fig. 2:**
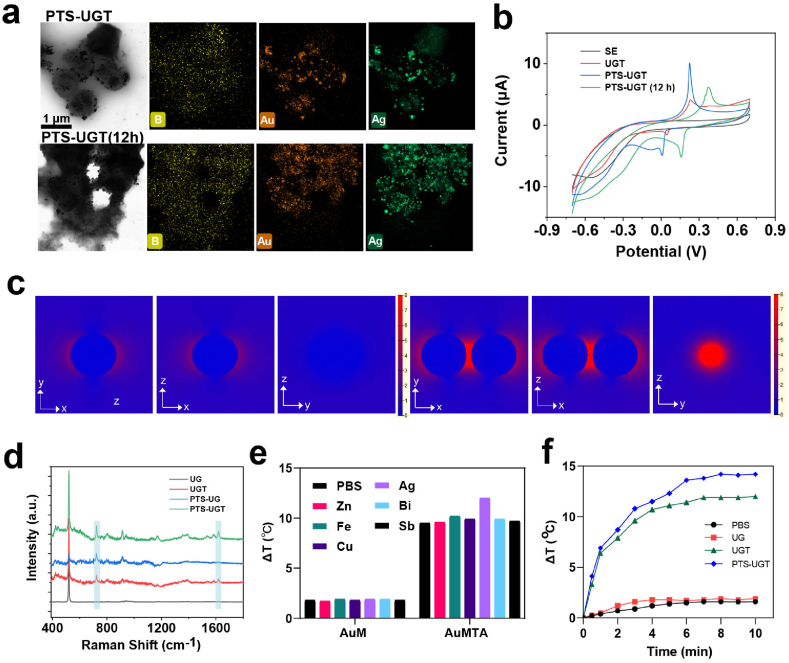
(a) Elemental mapping image of PTS-UGT and PTS-UGT (12 h). (b) CV of SE, UGT, PTS-UG and PTS-UGT in PBS, respectively. (c) Electromagnetic field simulation of a single discrete gold nanoparticle and two gold nanoparticles by 3D-FDTD. (d) SERS of 10^−5^ M crystal violet on UG, UGT, PTS-UG and PTS-UGT modified surface, respectively. (e) Plot of temperature variation of hybrid bacteria prepared with different bimetallic ions under 808 nm laser irradiation (1 W/cm^2^, 10 min). (f) Temperature change plots of UG, UGT and PTS-UGT under 808 nm laser irradiation (1 W/cm^2^, 10 min).

The rational construction and the underlying optical and electrochemical properties of the bionic hybrid bacterial clusters were further analyzed using electrochemistry, laser confocal microscopic Raman spectroscopy and near-infrared thermometry [[52], [53], [54]]. It was found that PTS-UGT would grow with the prolongation of the co-assembly time of each component (up to 12 h) due to the strong hydrogen bonding, coordination and hydrophobic interactions, which induced the growth of PTS as a full-coverage stacking on the surface of the bacterial layer (Fig. 2a). The morphology with bacterial clusters remains under acidic conditions, which may be conducive to the material's more stable physical and chemical properties of optical/electronics and regulation of biological effects on cells/bacteria (Fig. S10).

The cyclic voltammetry (CV) was used to explore the electronic transfer properties (Fig. 2b). According to the peak size, position and slope of the CV graph, compared with UGT and PTS-UGT (12 h), UGT and PTS co-assembled for 4 h to form properly bridged PTS-UGT, which showed better electrochemical activity. This may be due to the formation of a better electronic network transmission channel and conductivity, which is more conducive to the transfer and acquisition of reaction electrons. This should also be more conducive to clearing the inflammatory state of oxidative stress in organisms and exerting the immunomodulatory function of bacterial-specific proteins, which is in line with our current rational design of multiplexed and aggregation-enhanced hybrid bacterial clusters.

In addition, the electromagnetic field around gold nanoparticles is simulated using the 3D finite-difference time-domain (3D-FDTD). The simulation results show that compared to a single discrete gold nanoparticle, two gold nanoparticles have superior electric field strength when they are distributed in an aggregated manner (Fig. 2c). Next, the characteristic SERS peaks of each group after mixing with crystalline violet were analyzed accordingly. Compared with other groups, the PTS-UGT group showed the strongest Raman signal, which indicated that appropriate aggregation of hybrid bacteria clusters had the potential to enhance the intensity of localized surface plasmon resonance (Fig. 2d) [55].

In addition, the synergistic photothermal effect of bimetallic hybrid bacterial clusters by constructed by gold and a series of different metals was also explored. Although the simple bimetallic bacterial cluster (AuM) did not show an obvious temperature increase, after incubating it with TA to further reduce the metal component (AuMTA), each group showed a certain temperature increase under 808 nm laser irradiation, especially the AuAgTA group (i.e., UGT). Besides UGT and PTS-UGT, indicating slight bimetallic doping and aggregation enhanced photothermal effect, respectively. For example, for PTS-UGT, the temperature increased by 12.2 °C under the irradiation of 808 nm for 10 min (1 W/cm^2^) (Fig. 2e and f), and it has good photothermal stability (Fig. S11). Thus, this series of results of optical/electronic properties and photothermal effect further demonstrate the successful preparation of rationally designed biomimetic hybrid bacterial clusters, as well as the diagnostic and therapeutic prospects of optical and electrochemical combined bacterial immunotherapy.

### 3.3 Cell regulation ability of PTS-UGT

**Fig. 3:**
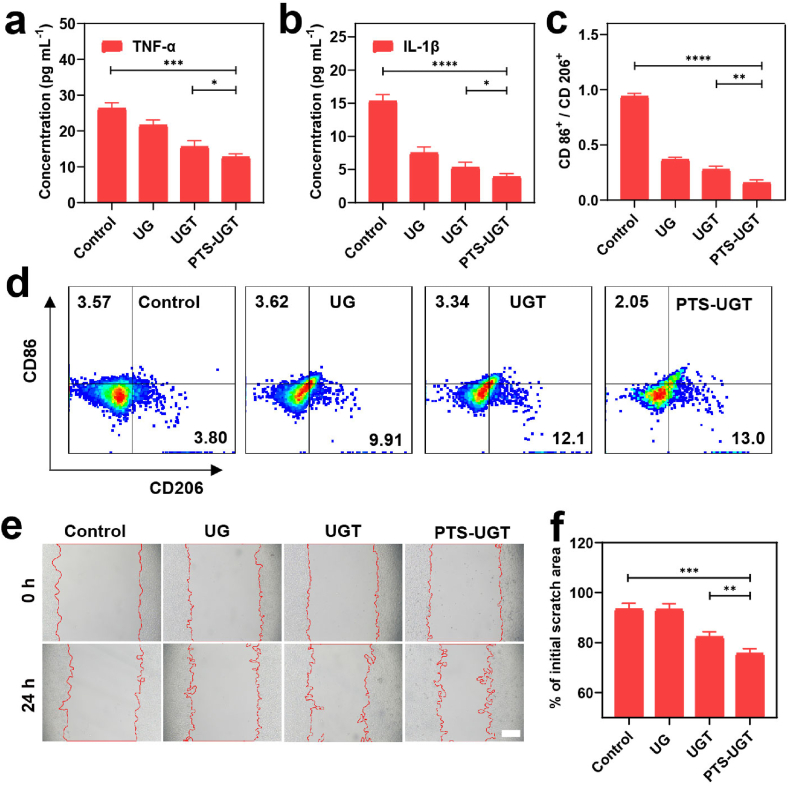
The concentration of TNF-α (a) and IL-1β (b) from RAW 264.7 cells after treatment with each group. (c) Statistical graph of CD86^+^/CD206^+^ ratio of RAW 264.7 cells. (d) The flow cytometry of RAW 264.7 cells treated with each group for the identification of CD206 versus CD86 phenotypic proteins. (e) Plot of 0 h and 24 h scratch assay of L929 cells after treatment with each group (Scar bar = 200 μm). (f) Statistical graph of cell migration rate of L929 cells after 24 h treatment. (∗p < 0.05, ∗∗p < 0.01, ∗∗∗p < 0.001, ∗∗∗∗p < 0.0001, n.s means no significance).

Macrophages and fibroblasts play an important role in wound healing. For example, inducing excessive M1 pro-inflammatory macrophages in the early stage of wound to polarize to M2 anti-inflammatory macrophages, which is beneficial to wound healing [56,57]. Macrophage phenotype regulation is closely related to material type, surface roughness and morphology [19]. The potential of SE to induce macrophage differentiation towards anti-inflammatory M2 type and to modulate fibroblast function has been reported [35,58]. Firstly, the toxicity to RAW 264.7 and L929 cells was determined by using CCK-8 kit. When the concentration of each group (OD_595_ = 0.3) was diluted 1-fold (1/2 eq), the cellular viability was more than 80 %, and no obvious hemolysis was observed in the samples of each group, indicating good biocompatibility (Fig. S12a–c). ROS levels and components related to the bacterial outer wall can affect the expression of inflammatory levels and macrophage polarization. For example, increased ROS oxidative stress or the widespread presence of LPS in Gram-negative bacteria can promote the expression of inflammatory macrophages. It is reported that Staphylococcus epidermidis bacteria contain unique proteins or lipoteichoic acid that can effectively reduce the expression of pro-inflammatory genes and reduce inflammatory cell infiltration, showing the induction of anti-inflammatory immunological characteristics [35,57]. So firstly, the cellular ROS levels were evaluated after incubation of each group with macrophage RAW 264.7. The results showed no significant increase in ROS levels in the UG, UGT, and PTS-UGT groups compared to the control group (Fig. S13). The corresponding hybrid bacterial cluster (PTS-UGT (*E. coli*)) prepared using Gram-negative bacteria (*E. coli*) as a template was used as a control, which had an effect on the ROS levels of macrophages RAW 264.7 (Fig. S14a and b). Next, the effect of PTS-UGT on the secretion level of macrophage inflammatory factor TNF-α and IL-1β and the content of cellular phenotypic protein markers (CD86, CD206) were determined. The TNF-α and IL-1β content was significantly reduced in the experimental groups, especially in the PTS-UGT group, compared with the control group (Fig. 3a and b). In the meantime, the ratio of CD86/CD206 was significantly reduced (Fig. 3c and d), which suggests that PTS-UGT can effectively promote macrophage polarization to anti-inflammatory M2 type, in virtue of the bacterial surface components, anti-inflammatory drug TA, and even the related bacterial co-aggregation biological effects and their spatial morphological characteristics (e.g., Morphology and larger size and rough surface) [19,59]. In addition, the cell scratch assays co-incubated with L929 cells showed that the PTS-UGT group could effectively promote cell migration compared with the control group (Fig. 3e and f)). In summary, bionic PTS-UGT has significant anti-inflammatory regulatory capabilities and the potential to promote wound healing.

### 3.4 In vitro antibacterial ability

**Fig. 4:**
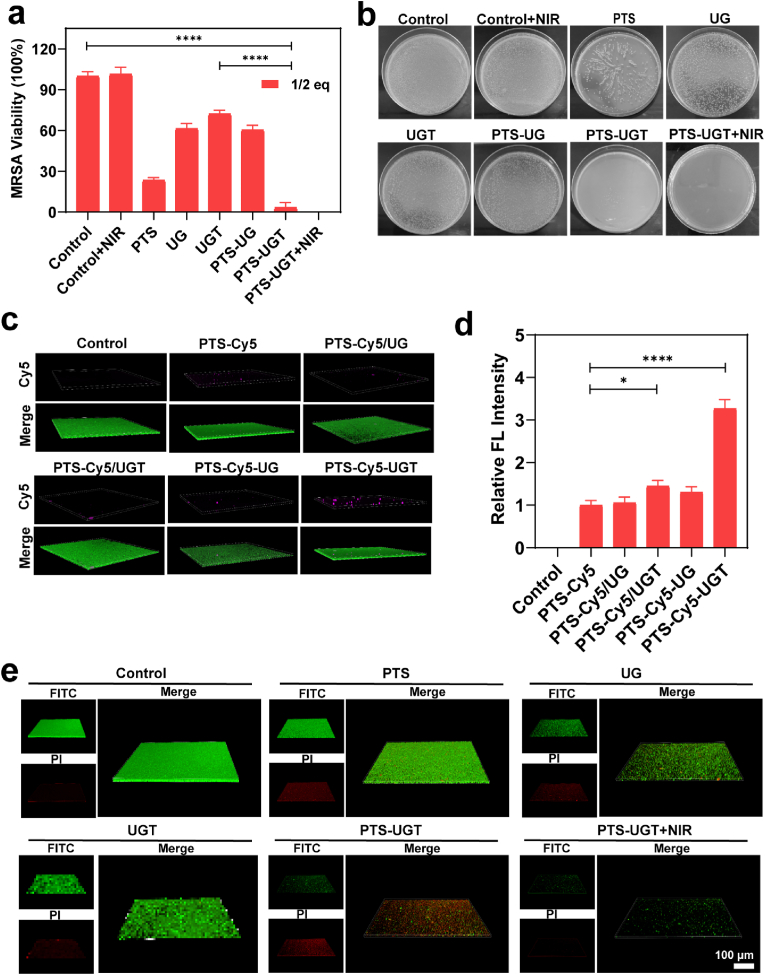
(a) Statistical graph of survival rates of MRSA after treatment with each group, (b) and the corresponding photographs of plate counts. (c) 3D CLSM images of the penetration and retention effect of each group in the bacterial biofilm. (d) Quantitative analysis of the Cy5 fluorescence intensity of each group in the bacterial biofilm. (e) Damage to bacterial biofilm assessed by live-dead staining for each group. (∗p < 0.05, ∗∗p < 0.01, ∗∗∗p < 0.001, ∗∗∗∗p < 0.0001, n.s means no significance).

As a skin commensal, SE plays an important role in the clearance of pathogenic bacteria such as Staphylococcus aureus (SA) by the metabolic regulation of skin-associated cells through secretions and its own associated components [34]. Here methicillin-resistant staphylococcus aureus (MRSA) was selected as a model for antibacterial studies. Under the aggregation and synergistic effect of antibacterial factors such as SE's own active ingredient, gold and silver antibacterial particles, tobramycin components and photothermal treatment, PTS-UGT + NIR demonstrated the most excellent antibacterial effects (Fig. S15). Considering the biocompatibility, PTS-UGT was chosen at 1/2eq concentration for detailed antibacterial effect study. At this time, the bactericidal efficiencies of PTS-UGT and PTS-UGT + NIR groups were 96.6 % and 100 %, respectively (Fig. 4a and b). It is worth mentioning that at this time, PTS-UGT also showed excellent killing effect on bacterial MDR.*E.coli*, showing broad-spectrum antibacterial potential (Fig. S16). Combined with PI staining and bacterial intracellular DNA leakage results (Fig. S17a and b), it can be further demonstrated that Au, Ag, especially PTS, plays a more dominant antibacterial role in antibacterial effects. In addition, the antibacterial component PTS on PTS-UGT is enriched in hybrid bacterial clusters and has a higher local concentration, which may also further contribute to the antibacterial effect similar to the behavior of mimetic host defense aggregation. The SEM images show that PTS-UGT still maintained hybrid bacterial morphology properties based on material biomodification strategies (Fig. S18).

It has been shown that SE modulates the downstream expression of the SA population sensing system and inhibits SA biofilm formation, virulence factor secretion and pathogenicity [60,61]. Thus, it is possible that under the synergistic effect of SE intrinsic inhibitory components and organic/inorganic nano-hybrid antibacterial components, the PTS-UGT and PTS-UGT + NIR group presented an biomimetic synthesis and optimization of the inhibitory effect on biofilm formation (Fig. S19), with biofilm formation inhibition rates of 69.6 % and 90.0 %, respectively.

In addition, the clearance efficacy of conventional antibacterial drugs (such as TOB) in mature biofilms is a challenge, because of poor drug retention and penetration capacity as influenced by the biofilm matrix. To check this, we first investigated the penetration of PTS-Cy5-UGT in MRSA bacterial biofilms. Compared to the control group, the PTS-Cy5-UGT group showed superior penetration and retention ability possibly due to their high drug coupling rate of PTS and the unique bacterial cluster morphology of larger size and rough surface (Fig. 4c and d). The live-dead staining and method was further used to evaluate the disruption of mature biofilm. Compared with other groups, the PTS-UGT group and the PTS-UGT + NIR group had better biofilm eradicate effects (Fig. 4e), with an eradication ratio of 55.15 % and 86.85 %, respectively. In addition, rystal violet staining results also showed that PTS-UGT and PTS-UGT + NIR had better anti-biofilm effect than other sample groups (Fig. S20). This is mainly attributed to the excellent multi-component antibacterial properties of PTS-UGT and its better enrichment effect in biofilms. However, it is also speculated that PTS-UGT has photoelectric activity and bacterial active ingredients that destroy biofilms, which may also induce the transfer of electrons from nearby pathogens to the extracellular space or even to the biofilm matrix, while aggravating the metabolic abnormalities of pathogens and the dysregulation of biofilm matrix secretion, which is beneficial to combat mature biofilms [[62], [63], [64], [65]]. This multicomponent hybrid bacterial cluster possesses excellent antibacterial properties in vitro, as well as significant inhibition, retention, and disruption effects on biofilms.

### 3.5 In vivo therapeutic evaluation

**Fig. 5:**
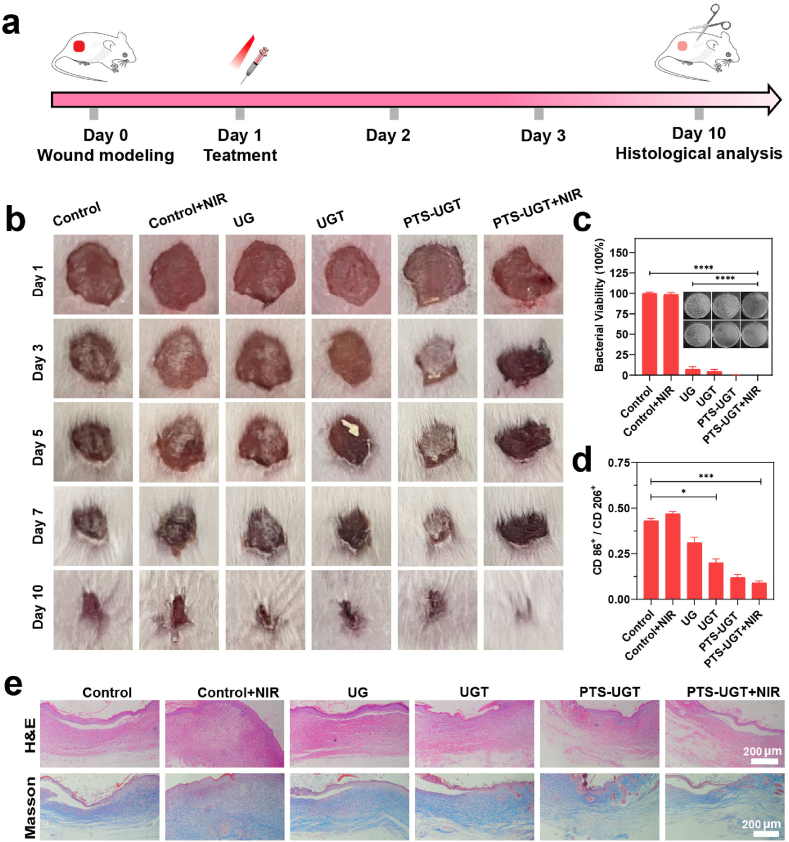
(a) Schematic of the process of bionic hybrid bacterial clusters for treating infected wounds in mice. (b) Photographs of wound healing under different samples. (c) Plate photographs and corresponding statistical histograms of bacterial survival on wounds in different treatment groups on day 3. (d) Statistical graph of macrophage phenotypic protein CD86^+^/CD206^+^ ratio of wound tissues treated with different samples on day 10. (e) Optical photographs of H&E staining and Masson staining of wound tissues treated with different samples on day 10. (∗p < 0.05, ∗∗p < 0.01, ∗∗∗p < 0.001, ∗∗∗∗p < 0.0001, n.s indicates no significant difference).

Based on the excellent modulation of cellular anti-inflammatory, pro-cell migration and significant antibacterial effects of PTS-UGT in vitro, a skin wound model of MRSA bacterial infection was established to evaluate its pro-healing efficacy in vivo. The treatment protocols were shown in Fig. 5a. Under 808 nm laser irradiation (1 W/cm^2^, 10 min), the temperature of the Control + NIR group slightly increased, while the PTS-UGT + NIR group increased by 10.2 °C (Fig. S21a). After a 10-day treatment assay, the wounds in the PTS-UGT and PTS-UGT + NIR groups exhibited a superior rate of healing compared to the Control and other groups (Fig. 5b–S21b), while mice in all groups show steadily gained weight (Fig. S21c). The wound bacterial load was assessed by plate counting method. Compared to the Control and Control + NIR groups, the UG and UGT groups exhibited moderate bacterial killing effect, while the PTS-UGT group and the PTS-UGT + NIR group exhibited excellent synergistic antibacterial effect with 100 % bactericidal rate (Fig. 5c).

In order to further verify the actual excellent treatment effect of PTS-UGT in vivo, after each group of samples was treated for 10 days, the infected wound tissues of each group were taken to analyze their inflammatory macrophage content and healing status. Flow cytometry results showed that the CD86/CD206 ratios in the PTS-UGT and PTS-UGT + NIR groups were significantly lower than those in the control group, indicating that the PTS-UGT group had higher levels of M2 anti-inflammatory and pro-repair macrophages at this time (Fig. 5d–S22). Correspondingly, H&E and Masson staining (Fig. 5e) showed that PTS-UGT, especially PTS-UGT + NIR, had less inflammatory infiltration, richer collagen deposition, and better tissue healing than the control group and other groups. In addition, the hearts, spleens, lungs and kidneys of mice in each group were collected for H&E staining. There are no significant differences among the groups, at the same time, combined with the results of metal accumulation in mouse tissues and serum biochemical and complete blood counts indices, it shows that the biomimetic polypeptide-based hybrid bacterial cluster, PTS-UGT, has good biosafety in vivo (Fig. S23–25).

**Fig. 6:**
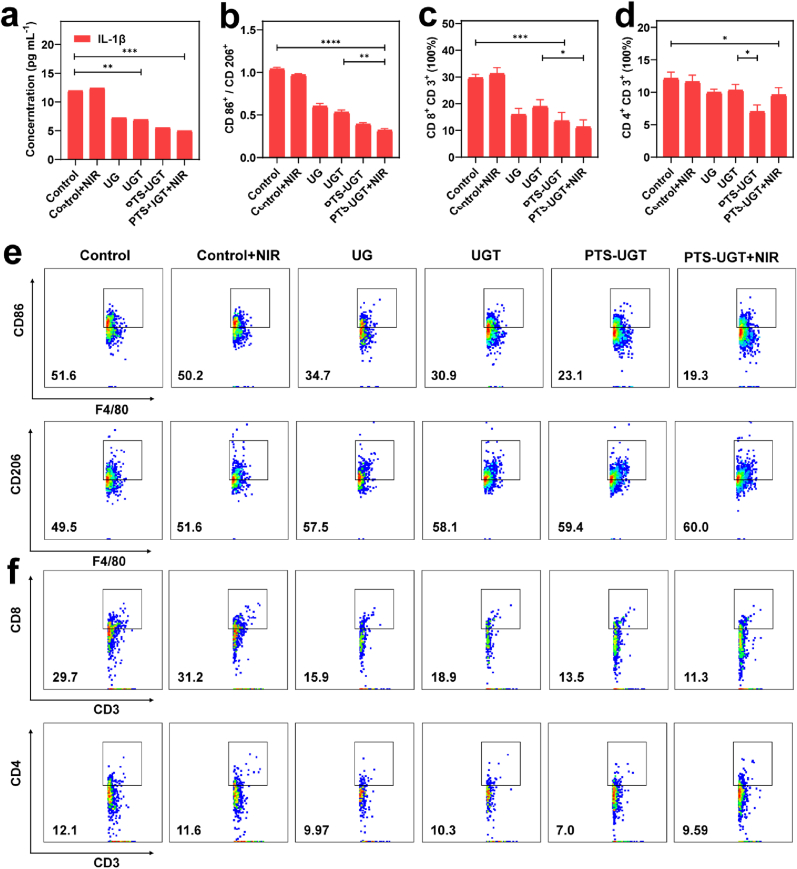
(a) IL-1β cytokine concentration in serum on day 3 of treatment in different treatment groups. (b) Statistical graph of macrophage phenotypic protein CD86^+^/CD206^+^ ratio of wound tissues treated with different samples on day 3. (c) Statistical plots of the CD8^+^/CD3^+^ ratio, (d) and the CD4^+^/CD3^+^ ratio of T-cell phenotypic proteins of wound tissues treated with different samples on day 3. (e) Flow charts for the identification of macrophage phenotypic proteins CD86 and CD206 of wound tissues treated with different samples. (f) Flow charts for the identification of T-cell phenotypic proteins CD8 and CD4 of wound tissues treated with different samples. (∗p < 0.05, ∗∗p < 0.01, ∗∗∗p < 0.001, ∗∗∗∗p < 0.0001, n.s means no significance).

To further analyze the antibacterial and anti-inflammatory mechanism of PTS-UGT, blood and infection site tissues of mice in the early stages (on day 3) were collected to identify inflammatory factor concentrations and immune cell differentiation [66,67]. Compared with the Control and Control + NIR groups, the inflammatory factor TNF-α, IL-6 and IL-1β decreased in the UG and UGT groups, which may be attributed to the antibacterial effect and the anti-inflammatory ability of the SE-related components with TA, whereas it significantly decreased in the PTS-UGT and PTS-UGT + NIR groups, which further demonstrated the excellent synergistic antibacterial and anti-inflammatory efficacy (Fig. 6a, S26). During wound healing, macrophages and T cells are closely related to bacterial clearance and inflammation relief in infected wounds. Macrophage phenotypic proteins CD86, CD206 and F4/80, and T-cell phenotypic proteins CD3, CD4 and CD8 were quantified by flow cytometry, respectively. The results showed that, compared with the Control and Control + NIR groups, CD86/CD206 in the PTS-UGT and the PTS-UGT + NIR group levels were significantly reduced, effectively transforming M1 pro-inflammatory to M2 anti-inflammatory macrophages (Fig. 6b–e). At the same time, compared with the control group, the number of cytotoxic T cells (CD4^+^/CD3^+^) and helper T cells (CD8^+^/CD3^+^) in the PTS-UGT group was also significantly reduced, which may indicate that the wound infection state is changing from the wound infection state to normal at this time (Fig. 6c, d, f). In conclusion, PTS-UGT could effectively reduce the level of inflammatory factors in bacterial-infected wounds by killing bacteria and regulating the polarization of macrophages to the M2 anti-inflammatory phenotype, avoiding the over-differentiation of pro-inflammatory immune cells, thus achieving effective reduction of inflammation and promoting the healing of infected wounds.

## 4 Conclusion

Inspired by the critical relationship between bacterial spatial aggregation and host-microbial interactions, the multiplexed biomimetic hybrid bacterial cluster PTS-UGT was constructed by a streamlined methodology combining stepwise bio-self-assembly with customized bioconjugation strategies. It shows Raman and electrochemical signal enhancement, as well as excellent anti-inflammatory, antibacterial, and pro-bacterial infected wound healing effects. Efficacy analyses reveal that PTS-UGT showed modulation of macrophage anti-inflammatory polarization, efficient penetration and disruption of biofilm, enhance the clearance of wound-infected bacteria, and significant attenuation of inflammatory immune cells (M1 macrophages, T cells) at wound sites.

This study aims to construct hybrid bacterial clusters with multiplexed components and assembly-enhanced efficacy. Thus, this paper employs a straightforward method of stepwise biological self-assembly and tailoring the conjugation for biological modification. We investigated polypeptide-mediated tailoring the conjugation that enables the enrichment of gold and silver nanoparticles on hybrid bacterial clusters for enhanced optical and electrochemistry activity properties, the accumulation of antibiotics for increased antibacterial efficacy, and the enhancement of immune modulation through the concentration of symbiotic bacterial proteins on these clusters. The goal is to realize an aggregation-enhanced therapeutic approach within a bioactive hybrid system mediated by nano-confinement of inorganic components and localized concentration increases of organic components. Although initial results are promising, further refinement in controlling molecular structures and assembly processes is necessary to further explore the finely spatiotemporal regulatory effects of hybrid bacterial materials on the body's immune system, such as T cells, DC cells, and B cells, in order to achieve superior enhancement multiplexed efficacy while minimizing interference from multiple pathways [68,69].

In summary, given the critical role of dynamically regulating bacterial aggregates in host-microbe interactions, we propose that future efforts could focus on optimizing molecular structures via bottom-up engineering strategies and integrating optical/electrochemical/acoustic microfluidic assembly modules. This integrated approach will enable precise control over the physicochemical properties and biological behaviors of hybrid bacterial aggregates, thereby providing an effective method to explore spatiotemporal regulation of biomimetic hybrid bacterial clusters in host body's defense and repair.

## CRediT authorship contribution statement

**Jiang Xiao:** Writing – original draft, Visualization, Methodology, Investigation, Formal analysis, Conceptualization. **Zhongquan Song:** Visualization, Methodology, Investigation, Formal analysis. **Xiangdong Lai:** Methodology, Investigation, Formal analysis. **Xiangyang Zhang:** Data curation, Formal analysis, Methodology. **Xiaohui Liu:** Writing – review & editing, Methodology, Funding acquisition. **Hui Jiang:** Writing – review & editing, Resources, Methodology, Investigation, Funding acquisition. **Minjie Li:** Writing – review & editing, Resources, Methodology, Investigation, Funding acquisition. **Xuemei Wang:** Writing – review & editing, Supervision, Project administration, Methodology, Investigation, Funding acquisition.

## Declaration of competing interest

The authors declare that they have no known competing financial interests or personal relationships that could have appeared to influence the work reported in this paper.

## Data Availability

Data will be made available on request.
