# Supplementary material for: Multiplexed polypeptide-based hybrid bacterial clusters by tailoring the conjugation for synergistic treatment of infected wounds
Source: Mater Today Bio. 2025 Jul 1;33:102040. doi: 10.1016/j.mtbio.2025.102040 (PMC12269884; doi:10.1016/j.mtbio.2025.102040)
Supplement: Multimedia component 1 [file mmc1.docx]

**Supplemental Information for**

**Multiplexed Polypeptide-based Hybrid Bacterial Clusters by Tailoring the Conjugation for Synergistic Treatment of Infected Wounds**

Jiang Xiao^a^, Zhongquan Song^c^, Xiangdong Lai^a^, Xiangyang Zhang^a^, Xiaohui Liu^a*^, Hui Jiang^a*^, Minjie Li^b*^, Xuemei Wang^a*^

^a^State Key Laboratory of Digital Medical Engineering, School of Biological Science and Medical Engineering, Southeast University, Nanjing, 210096, China

^b^Key Laboratory for Quality Evaluation of Bulk Herbs of Hunan Province, School of Pharmacy, Hunan University of Chinese Medicine, Changsha, 410208, Hunan (China)

^c^Zhongda Hospital, Medical School, Southeast University, Nanjing 210009, China

*Corresponding Author

101013182@seu.edu.cn (X.L.); sungi@seu.edu.cn (H.J.); mjlee@hnucm.edu.cn (M.L.); xuewang@seu.edu.cn (X.W., 0000-0001-6882-7774)

**Materials**

Chloroauric acid (HAuCl_4_ · 4H_2_O, 98.0%) were purchased from Sinopharm Ltd. (shanghai, China). Silver nitrate (AgNO_3_, 99.8%), Tannic acid (TA, 95%), n-Butylamine (99%), L-aspartic acid 4-benzyl ester (98%), Triphosgene (99%), Tetrahydrofuran (THF, Extra Dry, 99.7%), Dimethyl sulfoxide (DMSO, Extra Dry, 99.7%), Tert-butyl methyl ether (AR), N,N-diisopropylethylamine (99.5%), Tobramycin (95%) and 5-Formyl-2-thiopheneboronic acid (98%) were purchased from Energy chemical (Anhui, China). N-hexane (AR) and ethyl acetate (AR) were purchased from Aladdin (Shanghai, China). Sulfo-Cy5-NHS were purchased from Meilun Biotechnology (Dalian, China). Deionized ultrapure water (18.2 MΩ cm^−1^) was employed throughout the study. Bacterial Luria-Bertani (LB) nutrient medium was purchased from Sinopharm Ltd. (Shanghai, China). The reactive oxygen species (ROS) assay kit was purchased Solarbio Co. Ltd. (Beijing, China). The anti-CD86-FITC, anti-CD206-PE and anti-F4/80-APC were purchased from Thermo Fisher Scientific (California, USA). Calcein/PI Live/Dead Viability/Cytotoxicity Assay Kit was purchased from Beyotime Bioechnology Co., Ltd (Shanghai, China). Acridine orange, crystal violet and other reagents used in this study were purchased from Shanghai Chemical Reagents Company (Shanghai, China).

**Cell culture**

L929 and mouse macrophages (RAW 264.7) cells were obtained from the Cell Bank of Shanghai Institutes for Biological Sciences, Chinese Academy of Sciences. Cells were cultured with high-glucose Dulbecco’s modified Eagle medium (DMEM, containing 10% FBS and 1% penicillin-streptomycin) at a constant humidity and temperature of 37 °C incubator and 5% CO_2_.

**Bacterial culture**

Staphylococcus epidermidis and MRSA were culture with LB medium at 37 ℃ and harvested in the exponential growth period. Quantification of bacterial concentration could be achieved by measuring optical density (OD) at 595 nm

**Animal Expeiments**

All animal experiments in this study were conducted following the "Guide for the Care and Use of Laboratory Animals" and approved by the Ethical Committee at Southeast University (protocol number: 20240407001). Female KM mice weighing approximately 20 g were purchased from Nanjing Qinglongshan Animal Breeding Farm for the animal experiments.

**Instrumentation.**

Proton nuclear magnetic resonance (^1^H NMR) spectra were recorded on a BRUKER ARX400 MHz spectrometer. Chemical shifts (δ) were reported in the units of ppm and referenced to the proton impurities. The polymer solutions (~15 mg/mL) for ^1^H NMR tests were prepared by directly mixing and shaking at room temperature. JEM-2100 transmission electron microscope (TEM, JEOL, Japan) and scanning electron microscope (SEM, Zeiss Ultra Plus, Germany) were used to characterize the morphology. An ultraviolet-visible spectrophotometer (UV-vis, Thermo Evolution 220, USA) and a fluorescence spectrophotometer (FL, Shimadzu RF-5301 PC, Japan) was used to record the absorption and fluorescence spectra. Zeta potential and dynamic light scattering (DLS) experiments were carried out with Zetasizer Nano ZS (Malven, England). Energy-dispersive X-ray spectroscopy (EDS, Zeiss Ultra Plus, GER), X-ray photoelectron spectroscopy (XPS, ULVAC-PHI Quantera II, Japan) and Surface-enhanced Raman scattering spectroscopy (SERS, Renishaw inVia, UK) was used to analyze element type, element valence, and molecular structure, respectively. CHI 660E electrochemical workstation (CH Instrument, USA) was used for electrochemical measurement. Fluorescent imaging was acquired with a laser scanning confocal microscope (CLSM, Nikon Ti eclipse E2, Japan).

**Synthesis of BLA-NCA**

L-Aspartic acid-4-benzyl ester (2 g, 8.96 mmol) and triphosgene (0.97 g, 9.96 mmol) were placed in 30 mL anhydrous tetrahydrofuran. The mixture was stirred at 50 ℃ for 2 hours and then stirred overnight at room temperature. The solvent was removed by rotary evaporation under reduced pressure. The crude product of BLA-NCA was purified by three times recrystallization using a mixture of ethyl acetate and n-hexane 1:5, v/v). The resulting white powder product was obtained after vacuum drying (yield: 91%).

**Synthesis of PBLA**

BLA-NCA (2 g, 8.03 mmol) was dissolved in 20 mL of DMSO solvent, and the solution was purged with nitrogen gas for 5 minutes to remove oxygen from the solution. Then, a solution of DMSO (0.5 mol/L, 536 μL, 0.268 mmol) containing n-butylamine as initiator was added to the mixture at room temperature and allowed to react for 3 days. The reaction solution was then precipitated three times by adding it dropwise to ten times the volume of tert-butyl ether, followed by vacuum drying to obtain a white solid product of PBLA (yield: 93%).

**Synthesis of Cy5-Labeled PBLA (PBLA-Cy5)**

PBLA (0.1 g, 0.805 mmol) and N,N-diisopropylethylamine (50 μL) was dissolved in 5 mL of DMSO, followed by the addition of 1.2 equivalents of Sulfo-Cy5-NHS. The reaction mixture was left to react 36 h at room temperature. The reaction solution was then dialyzed in deionized water for 3 days (water changed three times daily). The resulting white powder product was obtained after freeze-drying (yield: 81%).

**Synthesis of PT and Cy5-Labeled PT (PT-Cy5)**

PBLA (0.5 g, 4.25 mmol) or PBLA-Cy5 was dissolved in 25 mL of DMSO, followed by the addition of 3 equivalents of Tobramycin. The reaction mixture was left to react 36 h at room temperature. The reaction solution was then dialyzed in deionized water for 3 days (water changed three times daily). The resulting white powder product was obtained after freeze-drying (yield: 42%).

**Synthesis of PTS and Cy5-Labeled PTS (PTS-Cy5)**

PT (20 mg, 0.161 mmol) or PT-Cy5 was dissolved in 2 mL of PBS, and then 2.5 equivalents of 5-formyl-2-thiophene boric acid dissolved in the mixed solution of DMSO and H_2_O (1:9, v/v) were added. The reaction mixture was left for 2 h at room temperature and was then dialyzed in deionized water for 3 days. The resulting product was obtained after freeze-drying (yield: 89%).

**Synthesis of UGT**

As described in the literature [1], the freshly cultured Staphylococcus epidermidis were resuspended in PBS, added with HAuCl_4_ solution at a final concentration of 150 μM and shaken for 4 h at 37 ℃. After centrifugation and washing with PBS twice, the sample was added with AgNO_3_ solution at a final concentration of 150 μM and continuously shaken for 4 h to obtain UG. To prepare UGT, the UG was added with TA solution at a final concentration of 150 μM and shaken for 4 h, then centrifuged and washed twice, and resuspended in PBS for storage.

**Synthesis of PTS-UGT and Cy5-Labeled PTS-UGT (PT-Cy5-UGT)**

A sufficient amount of PT or PT-Cy5 (final concentration = 1 mg/mL) and 5-formyl-2-thiophene boric acid (2.2 equivalents of PT or PT-Cy5) were mixed in PBS at room temperature for 2 h. Then UGT (final OD = 0.3) were mixed and shaken at room temperature for a certain time (2 h or 10 h), then centrifuged and washed twice to prepare PTS-UGT, which was resuspended in PBS for storage or dilute to different concentrations for later use (1 equivalents (eq), 1/2 eq, 1/4 eq). PTS-UG and Cy5-Labeled PTS-UG (PT-Cy5-UG) was prepared by similar method.

**Electrochemical analyses**

A three-electrode system was used for CV analysis. According to the literature [2], 8 μL of each sample solution was dropped on the glassy carbon electrode as a working electrode, and dried at 37 ℃. Platinum wire was used as a counter electrode and Ag/AgCl as a reference electrode. The scanning rate was 1 mV/s and was repeated for four cycles in the potential window from - 0.7 V to + 0.7 V.

**Raman spectroscopic analyses**

The bacterial modified materials were mixed with crystal violet at a final concentration of 10^-5^ M and dripped on the substrate. The SERS enhancement effect was detected under the laser excitation of 785 nm.

***In vitro* biosafety**

The CCK-8 assay kit was used to evaluate the cytotoxicity of the material on L929 and macrophage RAW 264.7 cells. For an example, RAW 264.7 cells were seeded onto a 96-well plate at a density of 10^4^ cells per well. After cell adhesion for 24 h, the cells were treated by different groups for 24 h (Control (PBS), UG, UGT, PT, PTS-UGT). Subsequently, the CCK-8 assays were conducted following the instructions of kit. The absorbance was measured at a wavelength of 450 nm using a microplate reader.

**Analysis of ROS level of macrophages**

Macrophage RAW 264.7 cells were inoculated onto a 6-well plate (10^6^ cells/well), and then were washed three times after 24 h incubation with different samples. The cellular ROS levels were measured on a fluorescence microscope according to the instructions of the ROS detection kit (DCFH-DA probe).

**Phenotypic regulation of macrophages**

The macrophage RAW 264.7 cells after 24 h incubation with different samples were washed and fixed, blocked with 5% BSA for 30 min, incubated with antibodies (anti-CD86-FITC, anti-CD206-PE, and anti-F4/80-APC) for 1 h. The phenotype of macrophage RAW 264.7 cells was analyzed by flow cytometry.

**Cell scratch test**

The L929 cells were inoculated in a 6-well plate and scratched and washed after the cells covered the bottom of the plate (a gap was made in the center of the well with a 200 μL pipette tip). Then it was replaced by a serum-free DMEM medium containing different sample groups, and observed and photographed under a microscope at different times.

**Bacterial killing effect *in vitro***

Different sample groups were incubated with freshly cultured MRSA at 37℃ for 24 h. Then, the appropriately diluted bacterial suspension of each group (100 μL) was spread on LB agar plates and cultured at 37℃ until mature colonies were formed, and then counted to evaluate the antibacterial properties of the materials.

**Bacterial morphology characterization**

The morphologies of bacteria after treatment with different samples were observed using SEM. Firstly, the bacterial suspension in different treatment groups was centrifuged, washed, and purified. Then, the bacterial samples were fixed with 2.5% glutaraldehyde for 2 h. Subsequently, a series of ethanol solutions with increasing concentrations were used for dehydration. Finally, the samples were prepared on silicon wafers to examine the morphology of the bacteria.

**Inhibition of biofilm formation**

The biofilm formation was assessed by fluorescent staining. Firstly, coverslips were placed in a 6-well plate, and MRSA suspensions treated with different sample groups were added to each well. After 48 hours of incubation, the coverslips were washed and stained with 0.01% acridine orange for 20 min. Finally, the excess dye was washed off, and the biofilm inhibition was evaluated by observing and photographing under a CLSM. Three-dimensional reconstruction was performed after photographing with the Z sequence.

**Penetration and Disruption of mature biofilms**

Initially, the LB culture containing MRSA was placed in a 6-well plate and incubated at 37 ℃ to form mature bacterial biofilm. Subsequently, different samples containing Cy5 dye-labeled polypeptide components were applied to each well. After 30 min, the supernatant was slowly aspirated and the biofilm were washed gently and stained with 0.01% acridine orange for 20 min. Finally, the excess dye was washed off, and the biofilm penetration effect was evaluated by observing and photographing under a CLSM.

Furthermore, different samples were applied to each well (Control (PBS), Control+NIR, PTS, PTS-UG, PTS-UGT and PTS-UGT+NIR,1/2 eq) and after overnight treatment, the supernatant was slowly aspirated and washed gently. Finally, the destruction of mature biofilm was observed under a CLSM according to the instructions of the Calcein/PI Live/Dead Viability/Cytotoxicity Assay Kit.

***In vivo* treatment of bacterial wound infections**

First, the mice were anesthetized, and a circular wound with a diameter of approximately 1 cm^2^ was created on the back. Then, MRSA (100 μL, 1×10^8^ CFU/mL) was implanted into the wound and allowed to infect for 12 h before conducting the treatment experiment. The mice with infected wounds were divided into six groups, including Control (PBS), Control+NIR, UG, UGT, PTS-UGT, and PTS-UGT+NIR, respectively. After different treatments (100 μL, 1/2 eq) on day 1, the wound size was periodically photographed, and the mice's weight was recorded up to 10 days. On day 3, wound fluid was collected from the mice for bacterial quantification using a culture plate, and blood samples were collected from the mice to measure the concentrations of IL-1β cytokines. In addition, wound tissues were collected and flow cytometry was used to evaluate the differentiation of macrophages and T cells. On day 10 the mice's wound skin was collected and flow cytometry was used to evaluate the differentiation of macrophages, and stained with hematoxylin-eosin (H&E) and Masson to evaluate the wound healing progress, besides the main organs were collected with H&E staining for the biosafety of PTS-UGT *in vivo*.

**Statistical analysis**

All statistical analyses were performed in GraphPad 9.0 (Prism) and Microsoft Excel. All statistical tests were specified in the figure legends. A significant p-value represents a significant difference set at *p < 0.05, **p < 0.01, ***p < 0.001, ****p < 0.0001, n.s means no significance.


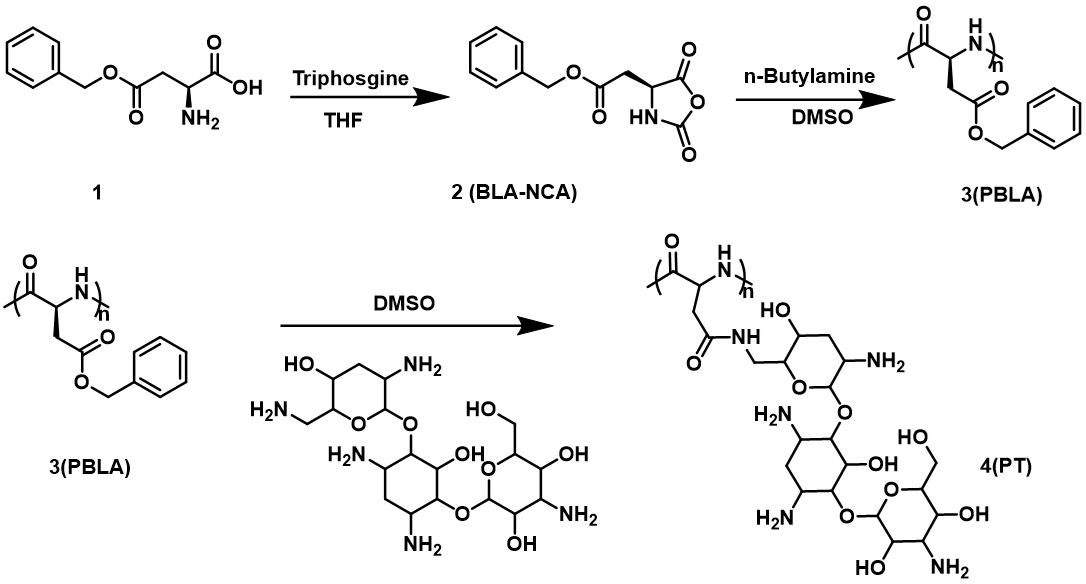


**Scheme S1** Synthetic route of PT.


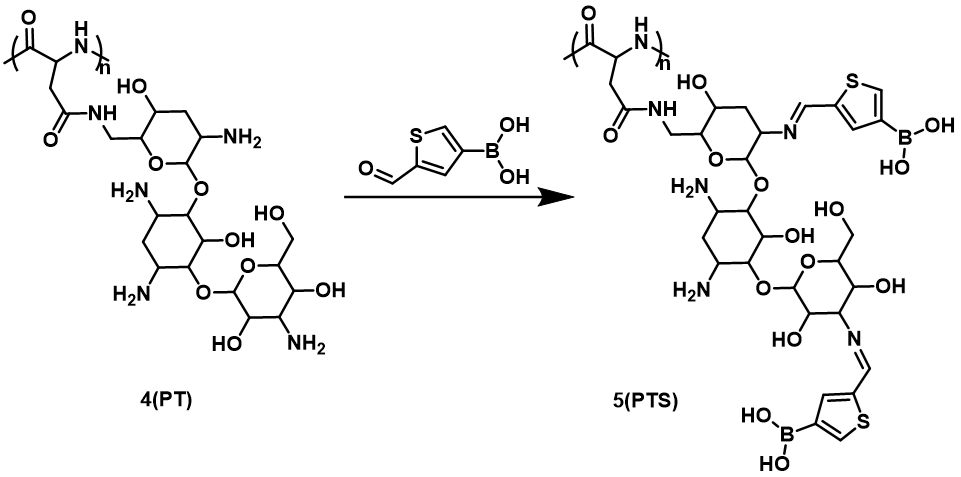


**Scheme S2** Synthetic route of PTS.

**
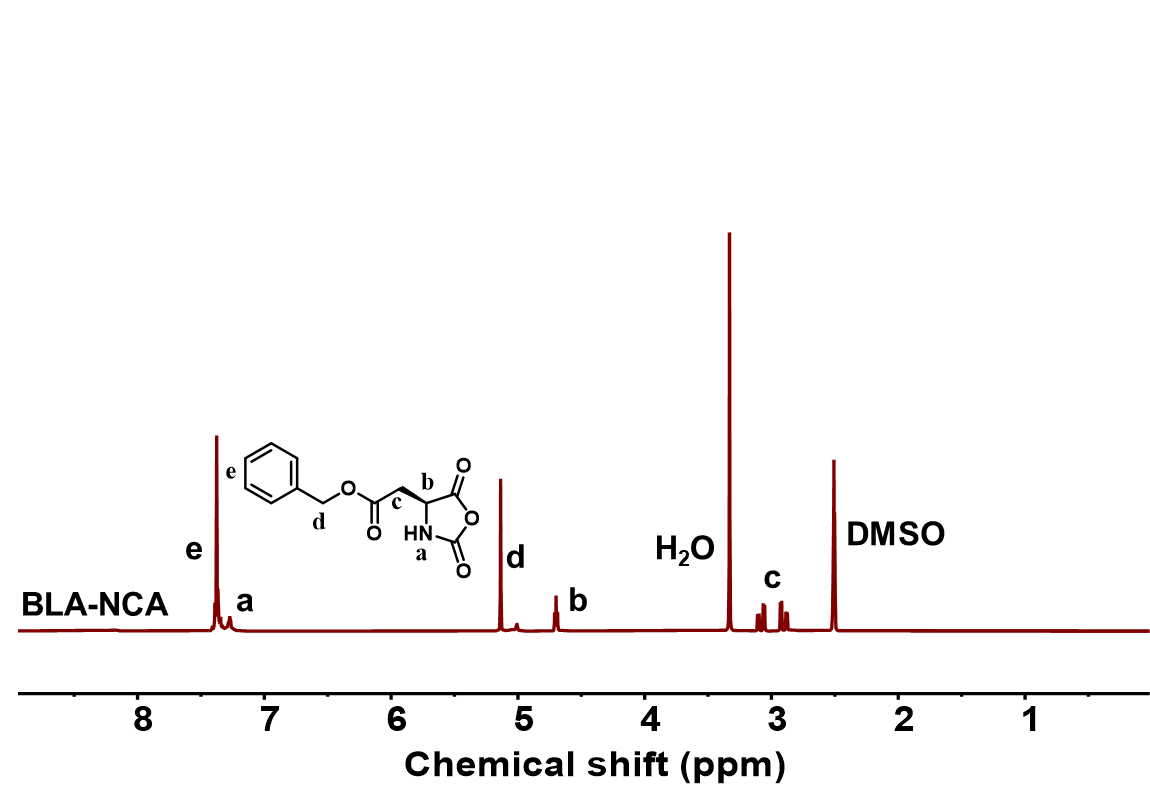
**

**Fig. S1** 1H NMR spectra of BLA-NCA in DMSO.


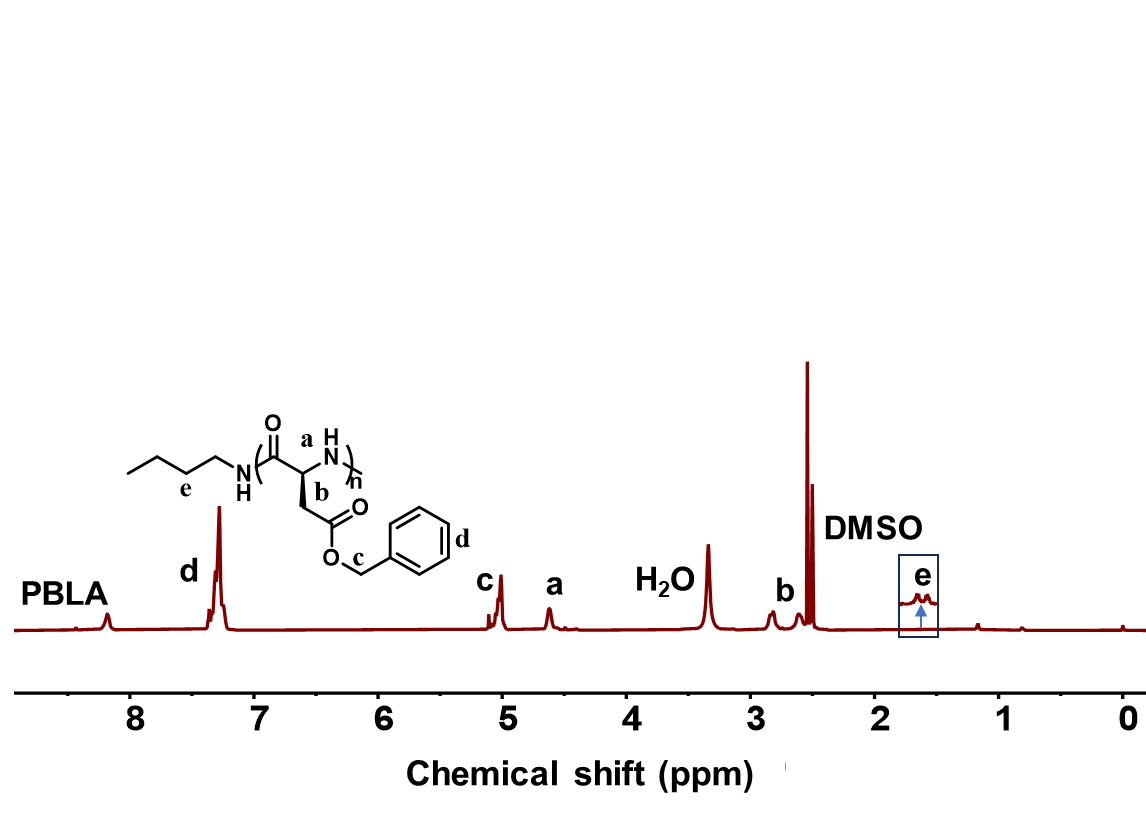


**Fig. S2** ^1^H NMR spectra of PBLA in DMSO.


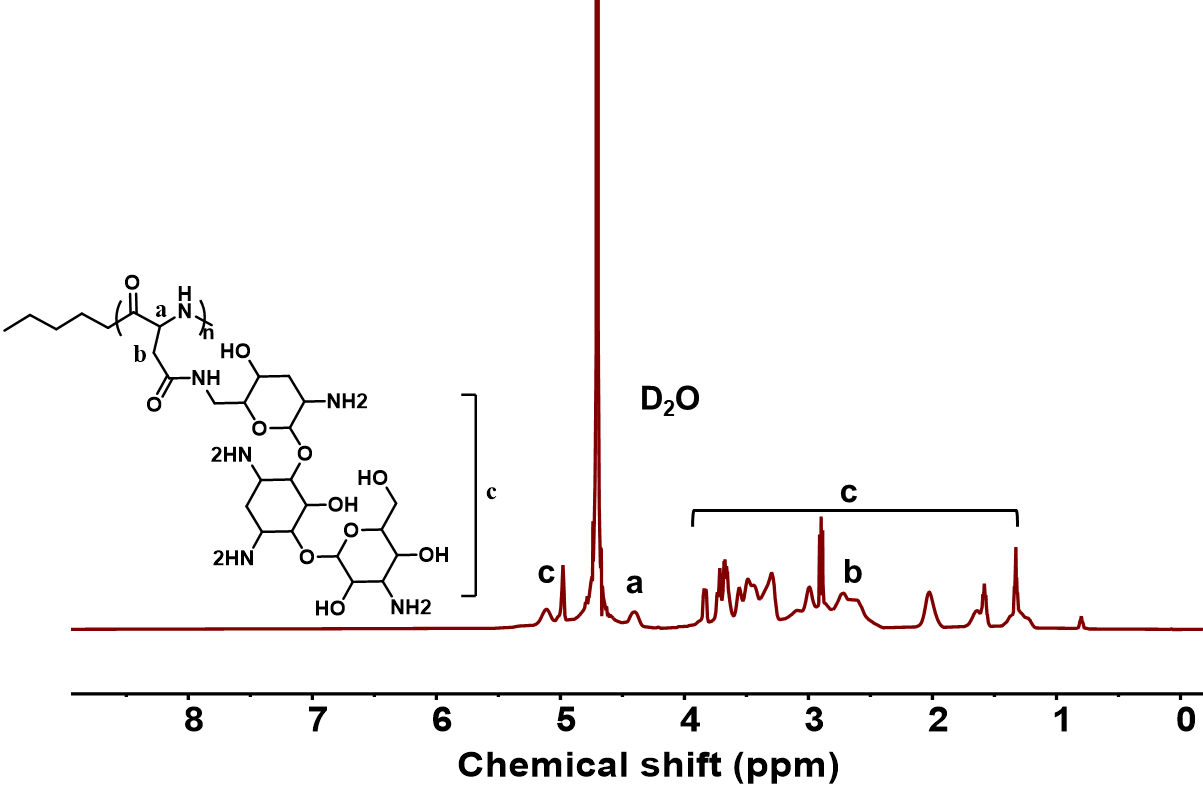


**Fig. S3** ^1^H NMR spectra of PT in D_2_O.


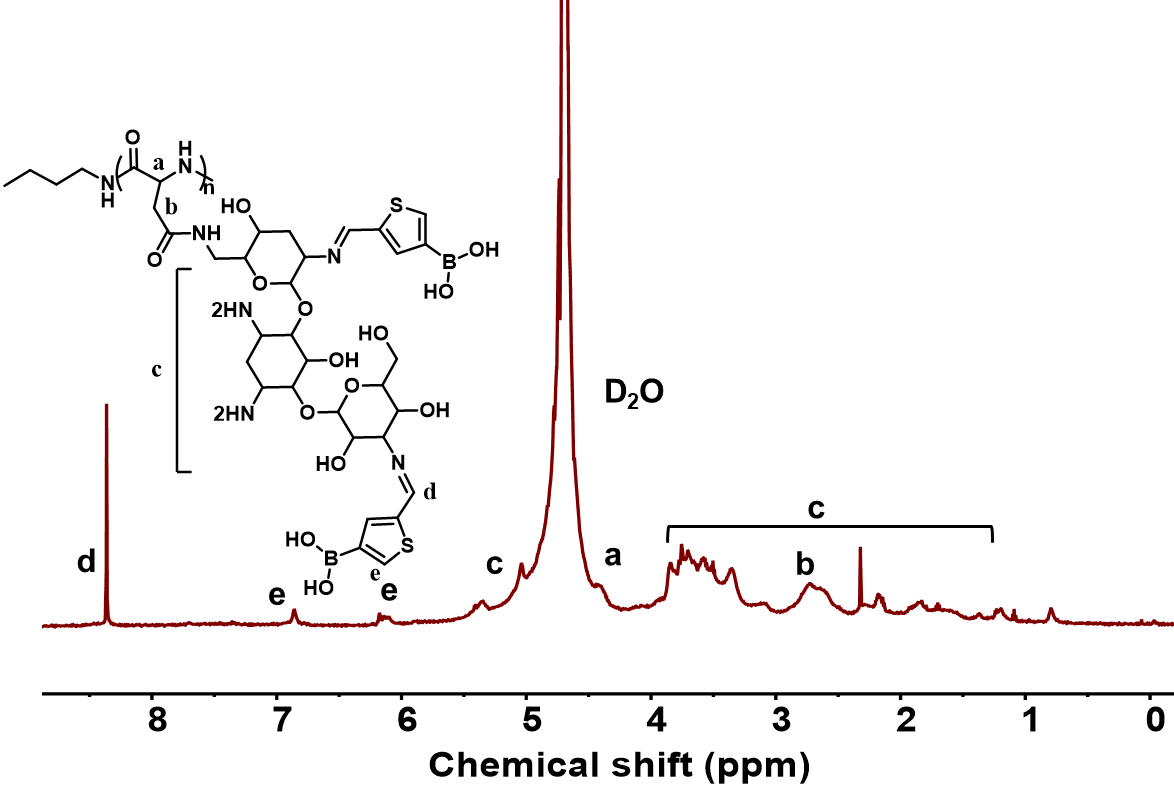


**Fig. S4** ^1^H NMR spectra of PTS in D_2_O.


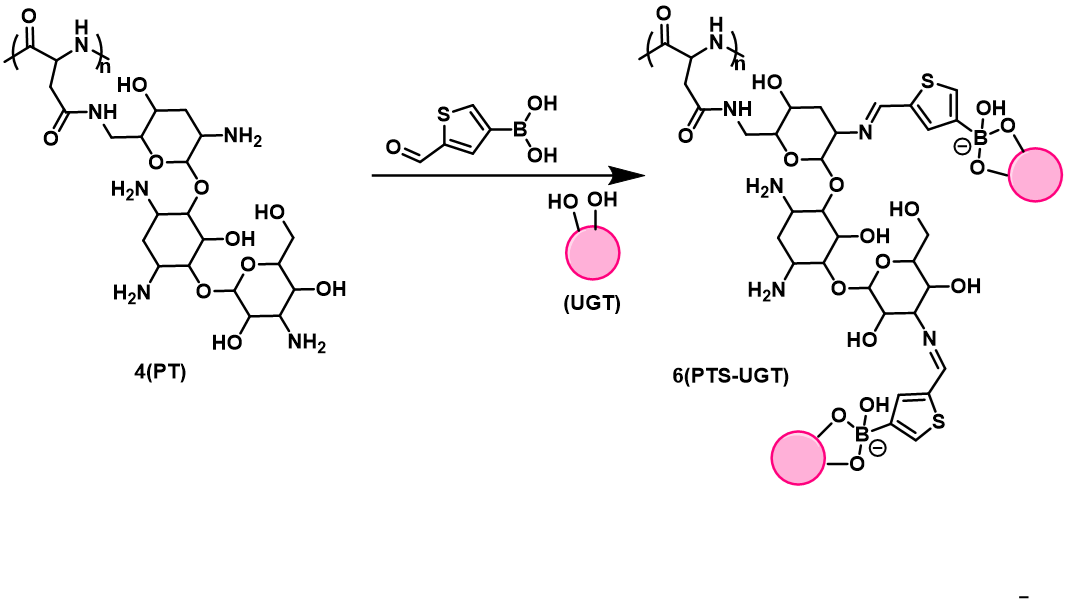


**Scheme S3** Synthetic route of PTS-UGT.


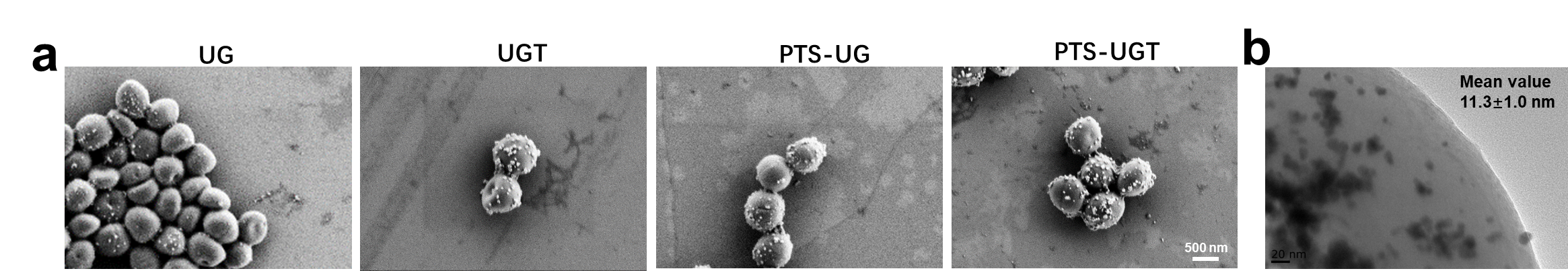


**Fig. S5** (a) SEM images of UG, UGT, PTS-UG and PTS-UGT.(b) TEM images of PTS-UGT and the size information of small Au and Ag nanoparticles on the surface.


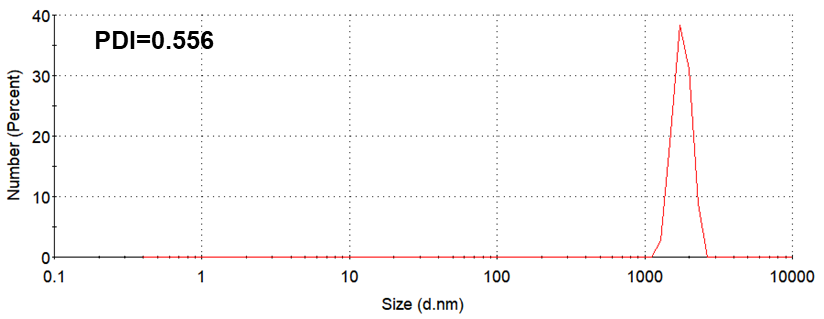


**Fig. S6** DLS origanal data of PTS-UGT.


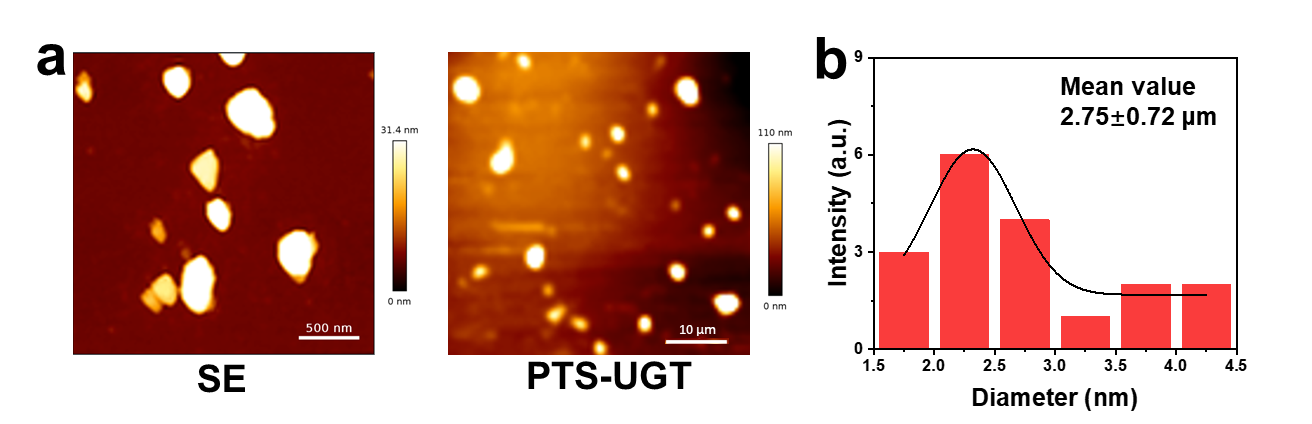


**Fig. S7** (a) Representative AFM images of SE and PTS-UGT. (b) statistical results of particle-size distribution of PTS-UGT.


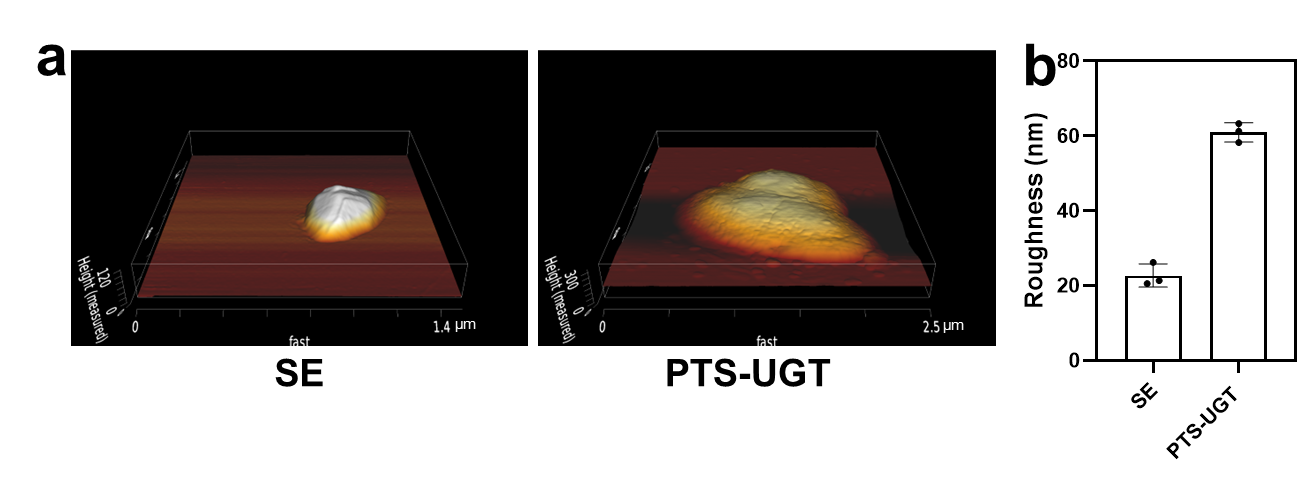


Fig. S8 (a) Representative 3D AFM images of SE and PTS-UGT and (b) the corresponding surface roughness.


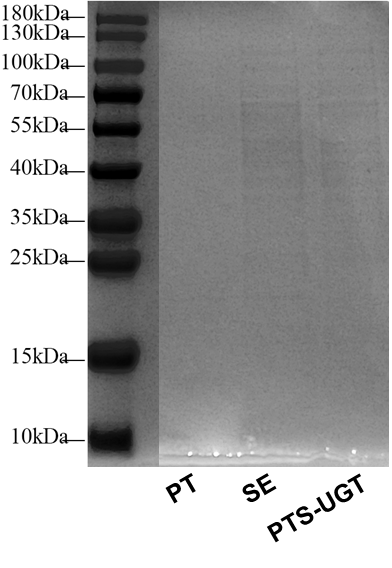


**Fig. S9**. Protein analysis of PT, SE and PTS-UGT.


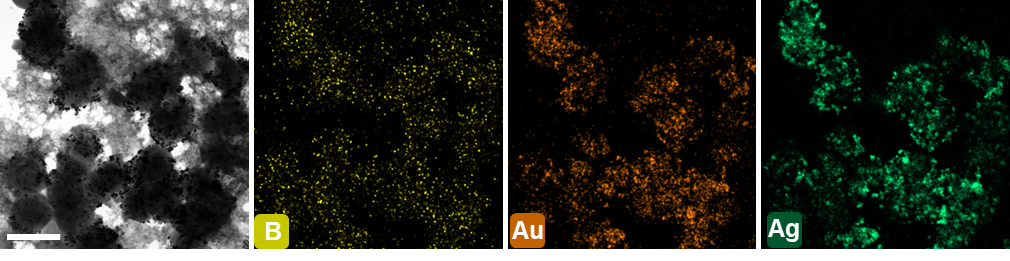


**Fig. S10** Elemental mapping images of PTS-UGT (pH = 5.5, 12 h, Scar bar = 1 μm).


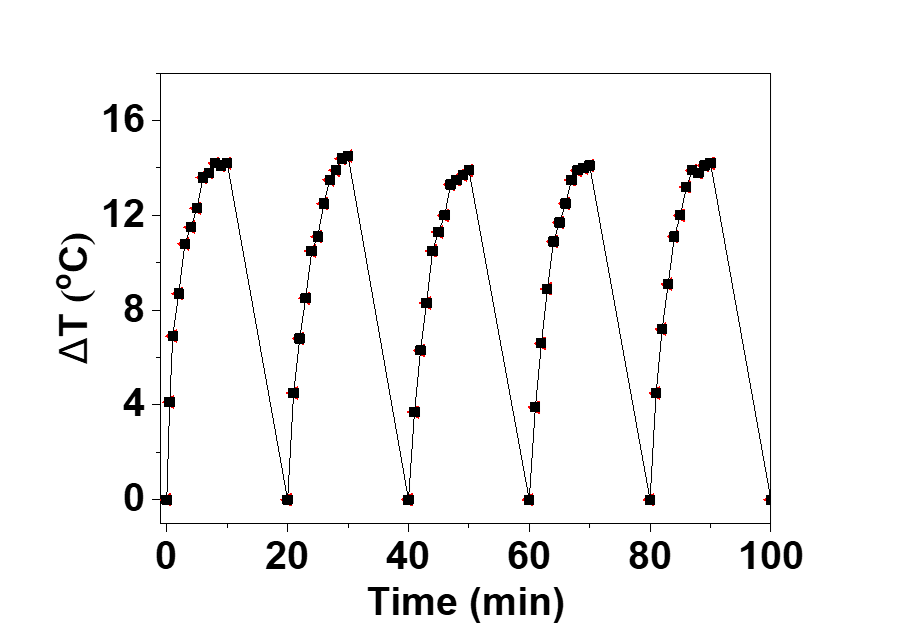


**Fig. S11** Long-term thermal cycle curve of PTS-UGT.

**
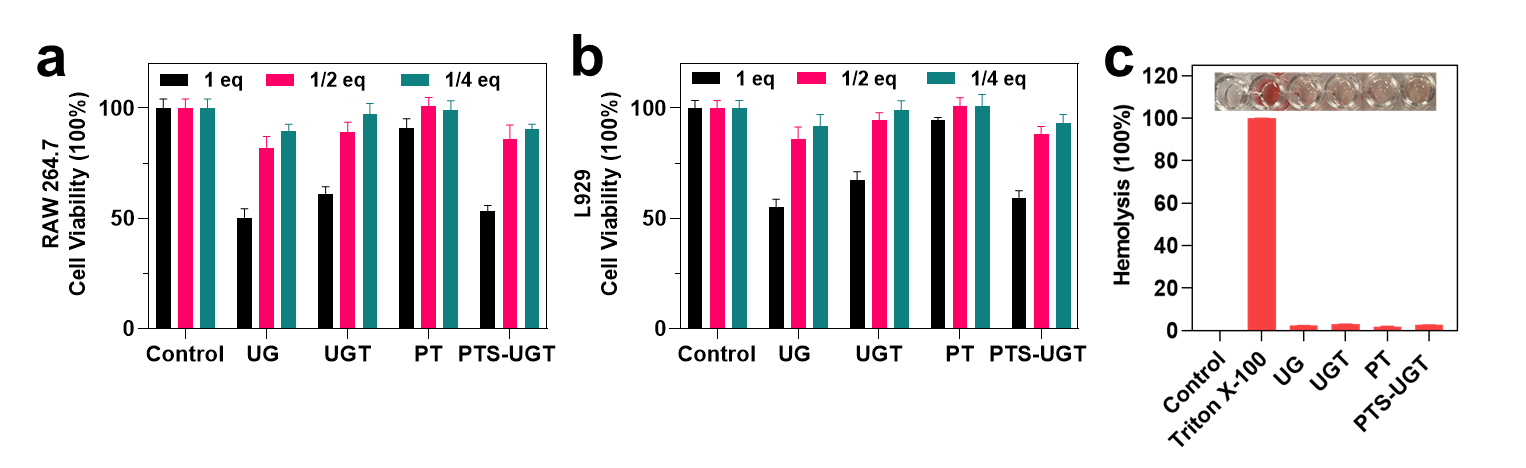
**

**Fig. S12** Cell viability of (a) RAW 264.7 cells and (b) L929 cells after treatment with different concentrations of samples. (c) Hemolytic analysis after different treatment.


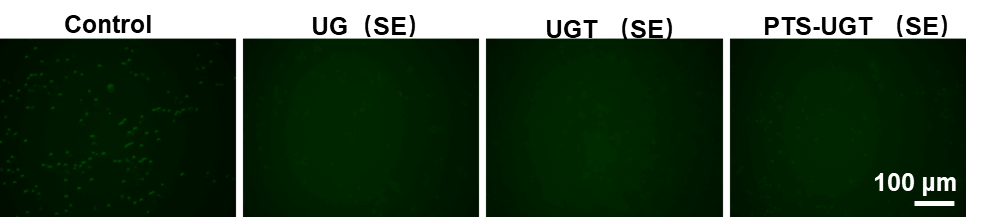


**Fig. S13** ROS levels in macrophage RAW 264.7 cells after different treatment.


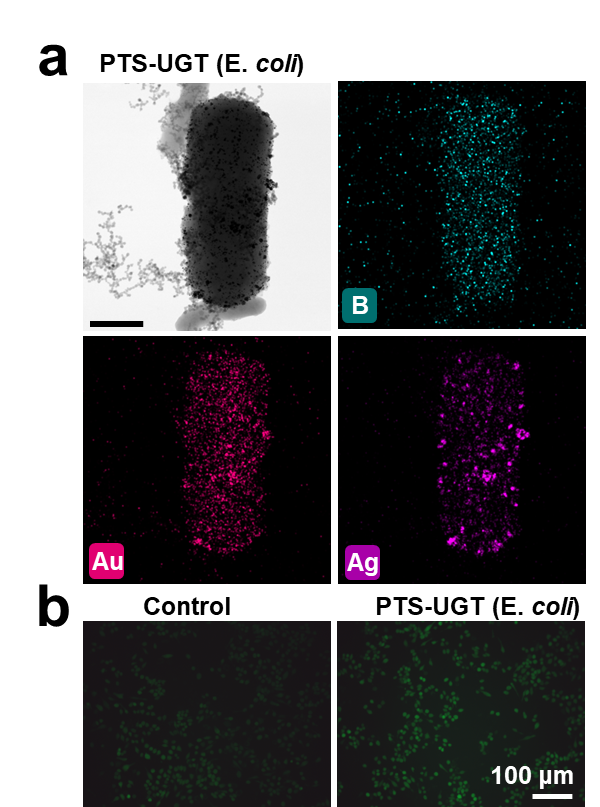


**Fig. S14** (a) Elemental mapping images of PTS-UGT (E. *coli*) (Scar bar = 500 nm). (b) ROS levels in macrophage RAW 264.7 cells after different treatment.

**Fig. S15** Bacterial viability after treatment with different concentrations of samples.


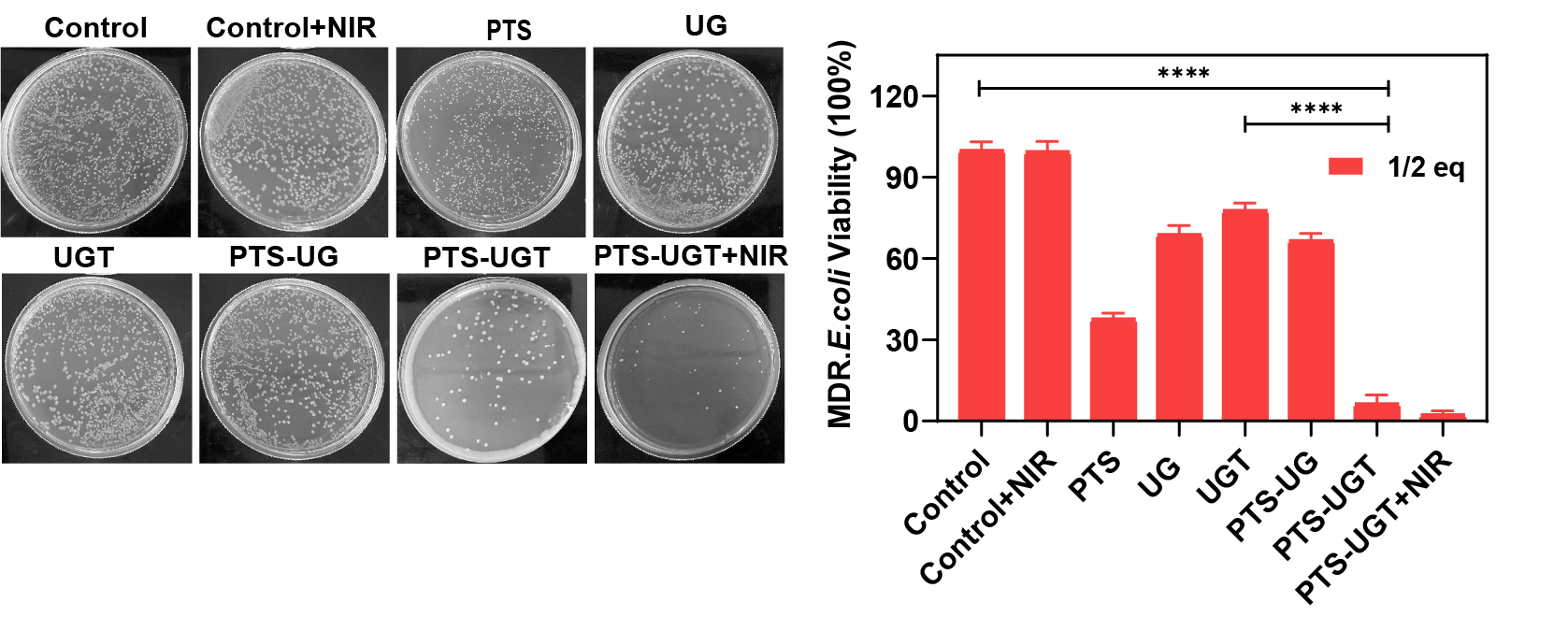


**Fig. S16** (a) photographs of plate counts of MDR.E.coli after treatment with each group, (b) and the .corresponding Statistical graph of survival rates.


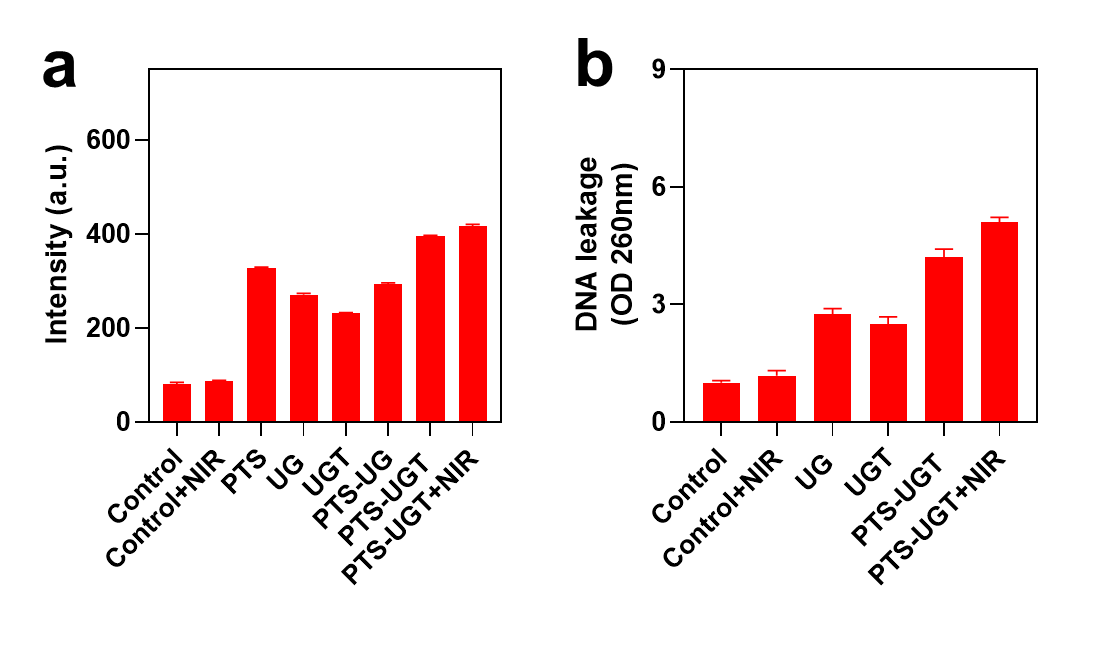


**Fig. S17** Determination of MRSA bacterial membrane permeability by propidium iodide (PI) staining (a) and MRSA bacterial DNA leakage (b) after different sample treatment.


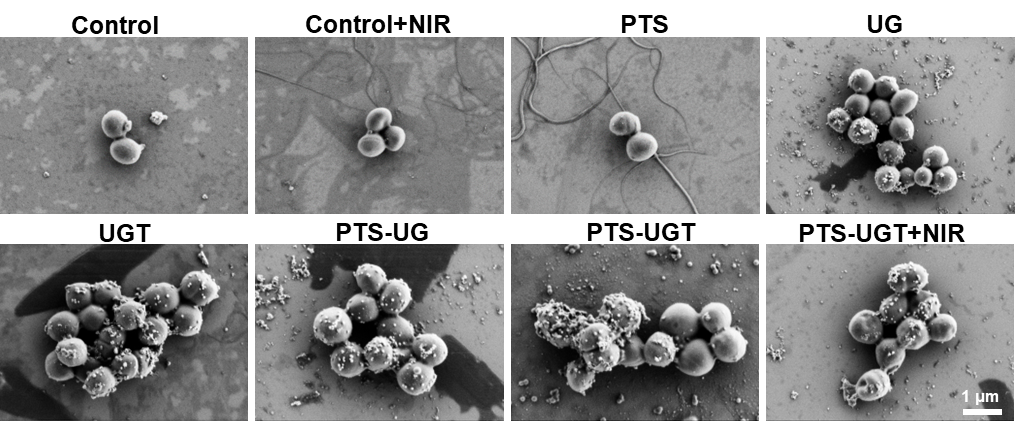


**Fig. S18** SEM images of MRSA after different treatment.


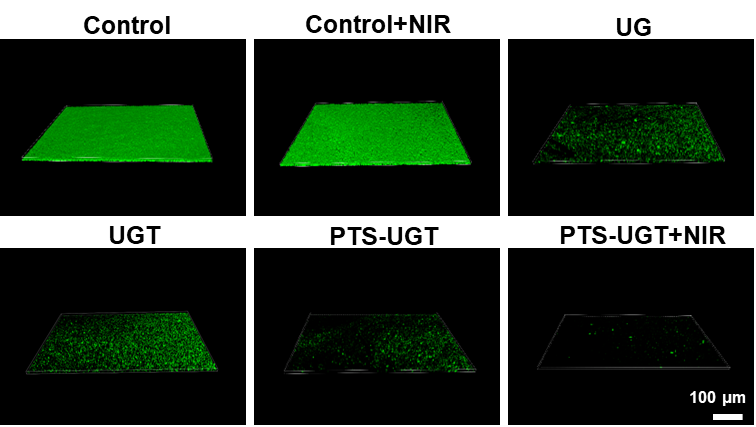


**Fig. S19** 3D CLSM images of MRSA biofilm labeled with acridine orange after different treatments.


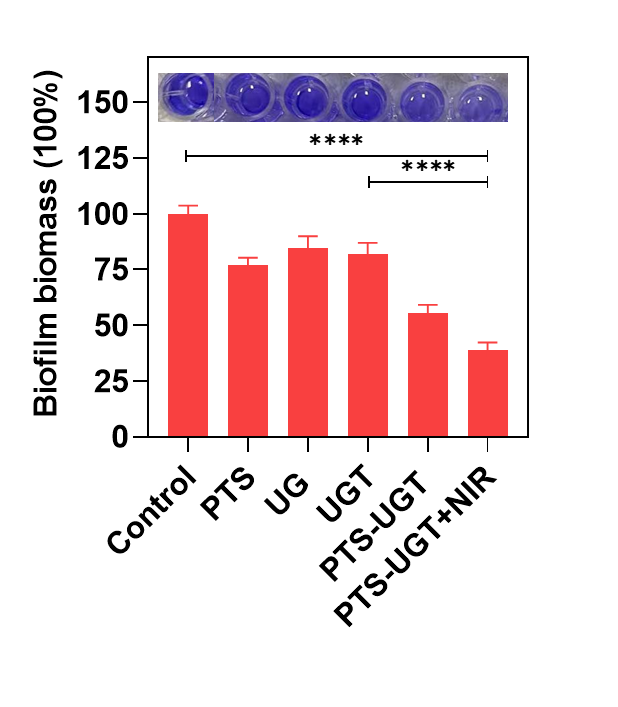


**Fig. S20** The relative biofilm biomass measured with crystal violet staining biofilms after different treatments.


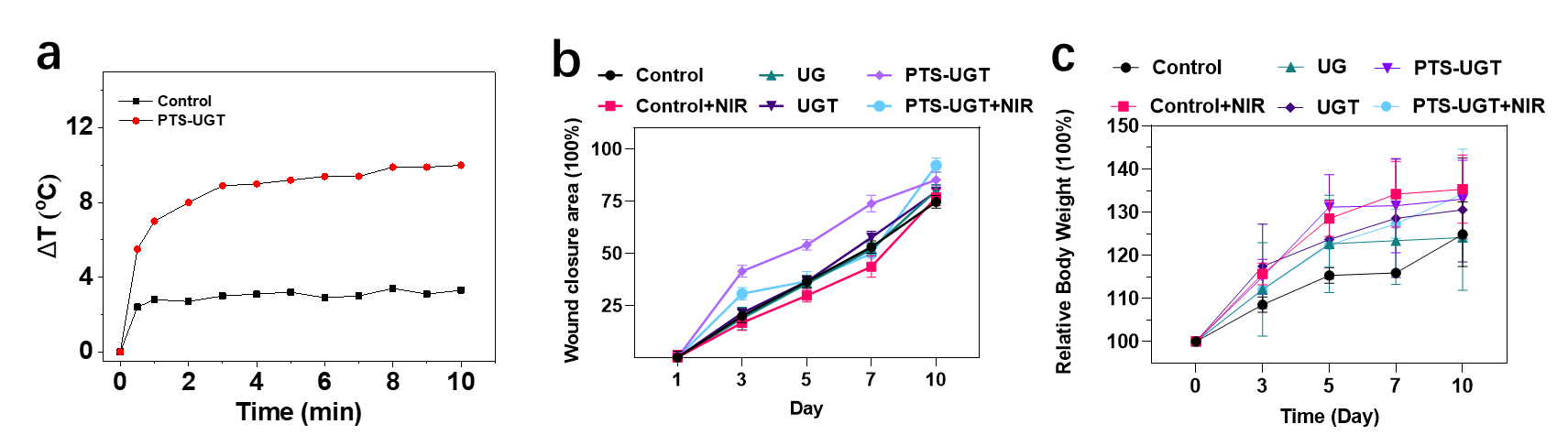


**Fig. S21** (a) Photothermal effect of mice under 808 nm laser illumination. (b) The wound closure area variation of mice after different treatments. (c) The body weight variation of mice after different treatments.


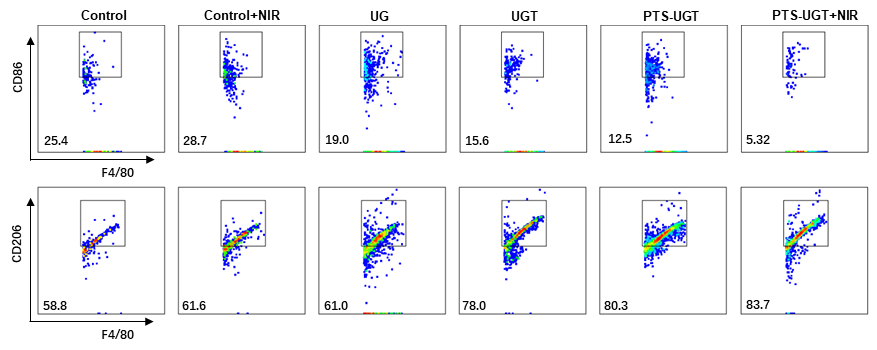


**Fig. S22** Flow charts for the identification of macrophage phenotypic proteins CD86 and CD206 of wound tissues treated with different samples on day 10.


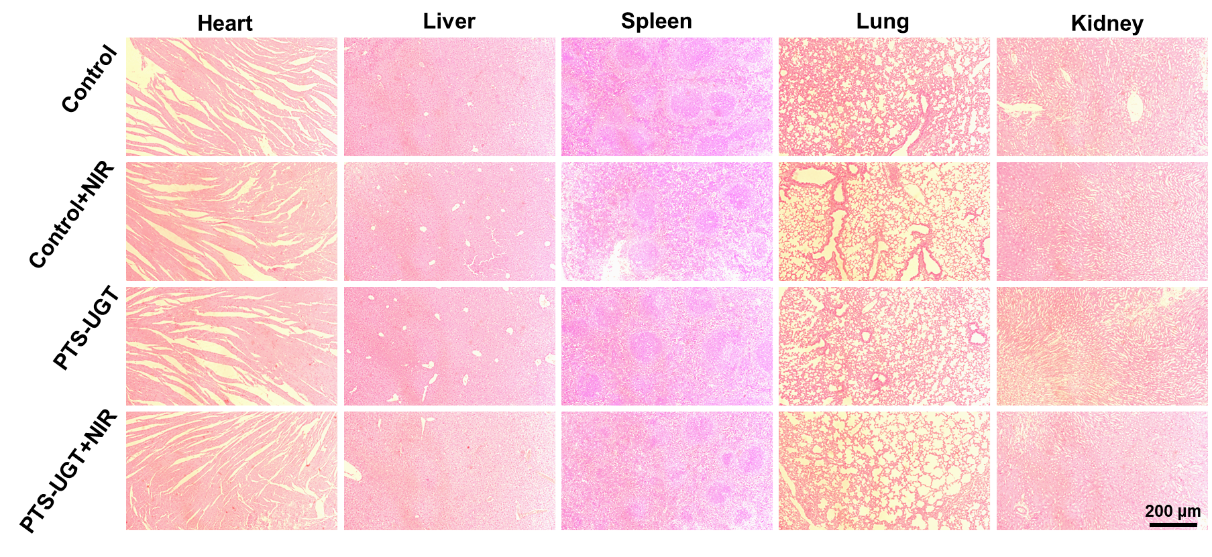


**Fig. S23** H&E-stained tissue slices of major organs (heart, liver, spleen, lung, and kidneys) excised from mice after different treatments.


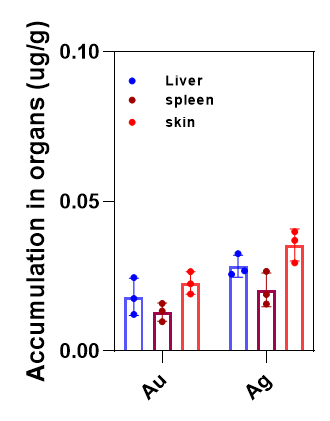


**Fig. S24** Gold/silver accumulation in organs (liver, spleen and skin) of PTS-UGT measured by ICP-MS.


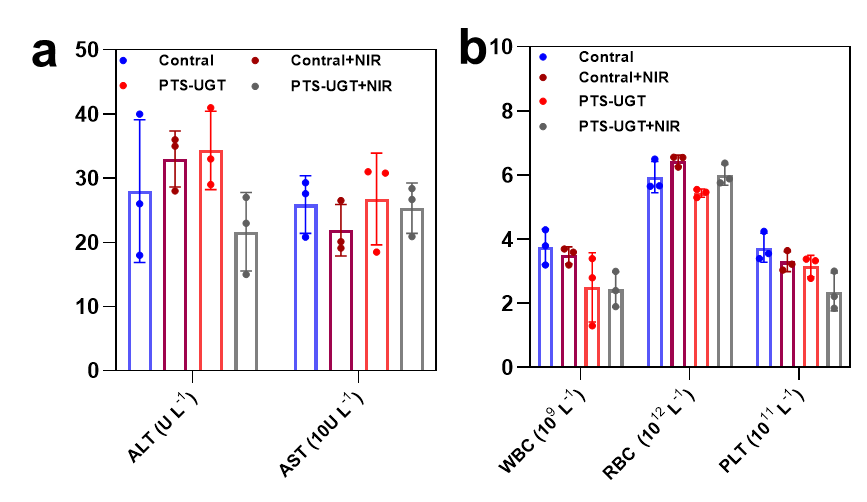


**Fig. S25** serum biochemical (a) and complete blood counts (b) indices of mice in different treatment groups.


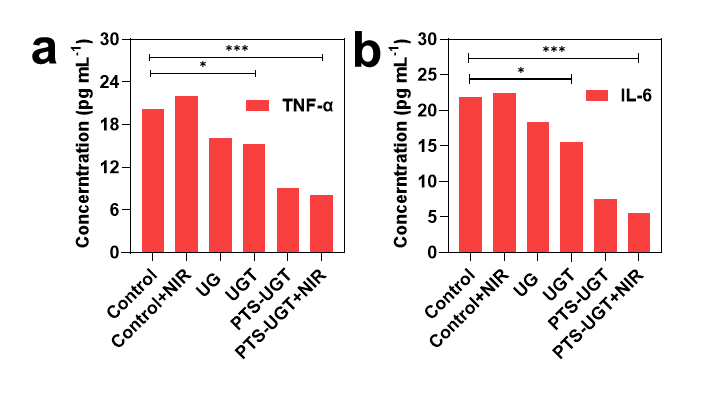


**Fig. S26** TNF-α (a) and IL-6 (b) cytokine concentration in serum on day 3 of treatment in different treatment groups.

**Table S1** Element atomic percentage determined by EDS and ICP-MS of Au and Ag in PTS-UGT.

| Element | AN | series | Net | [at.%] | μg/10^8^CFU |
| --- | --- | --- | --- | --- | --- |
| Au | 79 | L-series | 3564.27 | 20.6 | 2.13 |
| Ag | 47 | L-series | 12151.9 | 79.4 | 3.15 |

**References**

[1] J. Liu, Y. Zheng, Z. Hong, K. Cai, F. Zhao, H. Han, Microbial synthesis of highly dispersed PdAu alloy for enhanced electrocatalysis, Sci. Adv. 2 (2016) e1600858. https://doi.org/10.1126/sciadv.1600858.

[2] Q. Liu, Q. Ding, W. Xu, Y. Zhang, B. Zhang, H. Yu, C. Li, J. Zhang, Z. You, R. Tang, D. Wu, C. Zhao, Y. Cao, W. Lu, F. Li, H. Song, Engineering cell-electrode interfacial electron transfer to boost power generation of electroactive biofilm, Nano Energy 117 (2023) 108931. https://doi.org/10.1016/j.nanoen.2023.108931.
